# Improving Species Identification of Ancient Mammals Based on Next-Generation Sequencing Data

**DOI:** 10.3390/genes10070509

**Published:** 2019-07-05

**Authors:** Tian Ming Lan, Yu Lin, Jacob Njaramba-Ngatia, Xiao Sen Guo, Ren Gui Li, Hai Meng Li, Sunil Kumar-Sahu, Xie Wang, Xiu Juan Yang, Hua Bing Guo, Wen Hao Xu, Karsten Kristiansen, Huan Liu, Yan Chun Xu

**Affiliations:** 1 Laboratory of Genomics and Molecular Biomedicine, Department of Biology, University of Copenhagen, DK-2100 Copenhagen, Denmark; 2 BGI-Shenzhen, Shenzhen 518083, China; 3 China National GeneBank, BGI-Shenzhen, Shenzhen 518083, China; 4 College of Wildlife Resources, Northeast Forestry University, Harbin 150040, China; 5 Key Laboratory of State Forestry and Grassland Administration (State Park Administration) on Conservation Biology of Rare Animals in The Giant Panda National Park, China Conservation and Research Center of Giant Panda, Dujiangyan 611830, China; 6 School of Future Technology, University of Chinese Academy of Sciences, Beijing 100049, China; 7 Heilongjiang Provincial Museum, Harbin 150001, China; 8 Forest Inventory and Planning Institute of Jilin Province, Changchun 130022, China; 9 College of Informatics, Huazhong Agricultural University, Wuhan 430070, China; 10 State Key Laboratory of Agricultural Genomics, BGI-Shenzhen, Shenzhen 518083, China

**Keywords:** species identification, BLAST, ancient DNA, next-generation sequencing

## Abstract

The taxonomical identification merely based on morphology is often difficult for ancient remains. Therefore, universal or specific PCR amplification followed by sequencing and BLAST (basic local alignment search tool) search has become the most frequently used genetic-based method for the species identification of biological samples, including ancient remains. However, it is challenging for these methods to process extremely ancient samples with severe DNA fragmentation and contamination. Here, we applied whole-genome sequencing data from 12 ancient samples with ages ranging from 2.7 to 700 kya to compare different mapping algorithms, and tested different reference databases, mapping similarities and query coverage to explore the best method and mapping parameters that can improve the accuracy of ancient mammal species identification. The selected method and parameters were tested using 152 ancient samples, and 150 of the samples were successfully identified. We further screened the BLAST-based mapping results according to the deamination characteristics of ancient DNA to improve the ability of ancient species identification. Our findings demonstrate a marked improvement to the normal procedures used for ancient species identification, which was achieved through defining the mapping and filtering guidelines to identify true ancient DNA sequences. The guidelines summarized in this study could be valuable in archaeology, paleontology, evolution, and forensic science. For the convenience of the scientific community, we wrote a software script with Perl, called AncSid, which is made available on GitHub.

## 1. Introduction

The unearthing of ancient samples is an essential activity to reconstruct the biodiversity and biogeography of the prehistoric world, facilitating further understanding of evolutionary history and the reformulation of modern phylogeny, taxonomy, and evolution-based theories. The genetic information embedded in DNA sequences has been well mined and interpreted for modern species. Mining the genetic information of ancient species is thus urgently needed to compare with those of modern species and harmonize evolutionary systems. However, many ancient specimens excavated from archaeological fieldwork often lack morphological characteristics due to their long-term preservation and exposure to unfavorable environmental conditions. As such, it is challenging to accurately identify the species of many ancient samples based merely on their morphology.

A study conducted by Higuchi et al. 35 years ago found that highly degraded DNA may survive for a long period of time, and could be isolated and sequenced from a small piece of the dried muscle of the extinct quagga (*Equus quagga*) that had died 140 years prior to their study [1]. This opened the door to the study of ancient DNA [2], which led to the exponential growth of ancient genome-wide sequencing over the past few years [3,4,5,6,7,8,9], especially for ancient humans. These ancient DNA sequences have provided a great deal of key information and direct evidence necessary to resolve long-standing questions in ecology-, evolution-, and disease-related research [10,11,12]. It also enables the DNA-based species identification of ancient specimens.

Presently, the most frequently used methods for species identification are genetic-based PCR amplifications followed by sequencing and BLAST search (https://blast.ncbi.nlm.nih.gov/). These techniques have been continuously developed and widely used in various fields, including forensic science, paleontology, archaeology, wildlife research, and bird strike investigations [13,14,15,16,17]. DNA barcoding has become one of the most promising methods for species identification since Hebert et al. [18] established the first barcoding system using a ~600 bp DNA fragment located in the mitochondrial gene cytochrome *c* oxidase I (*COI*). However, a 600-bp amplicon is too large to be amplified from DNA recovered from ancient samples because DNA fragments in ancient remains could have been highly degraded, and the average DNA length of these fragments is usually less than 100 base pairs [19]. Therefore, shorter DNA regions (less than 200 bp) that are easier to amplify and highly polymorphic to allow species identification are preferable. Although the success rate can be significantly improved by amplifying shorter amplicons, very short DNA fragments often fail to identify remains to the level of definitive species during the BLAST search [20]. Thus, the tradeoff between the amplicon size and the success of species identification is obvious. 

The use of small amplicons plus multiple loci seems to be a more reasonable way to satisfy this tradeoff. Several studies have proved that this combination can successfully identify ancient birds [14], ancient fish [21], and other ancient animals [16,22]. However, although the amplicons used in these studies were smaller than 100 bp, the PCR amplification often failed to produce products necessary to identify the species of many ancient samples, which may have resulted from the highly degraded endogenous DNA. Besides, it is also very challenging to perform multiple rounds of PCR amplification because the amount of endogenous DNA in ancient samples is generally low. Designing universal primers for small amplicons is also very difficult due to the lack of sufficient nucleotide variations, which makes this method more tedious and time-consuming for the identification of multiple species. 

Next-generation sequencing (NGS) is a strategy that generates reliable sequence data ranging from 10 to 300 nucleotides, and is thus suitable for sequencing highly degraded DNA [4]. However, there are several challenges in analyzing ancient DNA using this approach. Firstly, mapping short fragments often results in different outcomes when different similarity criteria, query coverages, and algorithms are used. Secondly, ancient DNA is often deaminated at the two termini that induce nucleotide changes of sequence data (C-to-T and/or G-to-A). The identification and filtration of such introduced erroneous nucleotides must be carefully performed in order to avoid lowering the reliability of species identification. Lastly, DNA isolated from ancient specimens is highly contaminated by bacterial, fungal, and other sources of DNA. Filtration is confined by the contradiction that improving the cleanness of the target sequence comes at the cost of the quantity of effective data. Blow et al. [23] developed a method to identify ancient remains by enriching the ancient DNA using emulsion PCR amplification followed by NGS technology. However, a large number of PCR cycles and the PCR bias can, to some extent, influence the downstream species identification. Skoglund et al. [24] developed PMDtools by using a postmortem degradation score (PMD score) to distinguish genuine ancient DNA from modern DNA contaminations. This method is very effective in filtering modern human DNA contamination from the endogenous ancient human DNA. However, for animals, contamination from modern human DNA induced during sample collection and lab works can be easily removed by mapping against the reference genome, and the chances of ancient animal DNA contamination with DNA from the same modern species is very low. Besides, ancient DNA enrichment and the separation of ancient endogenous DNA from modern-day contamination are an upstream analysis of the species identification. Therefore, a more effective strategy or guidelines are still needed.

**Figure 1:**
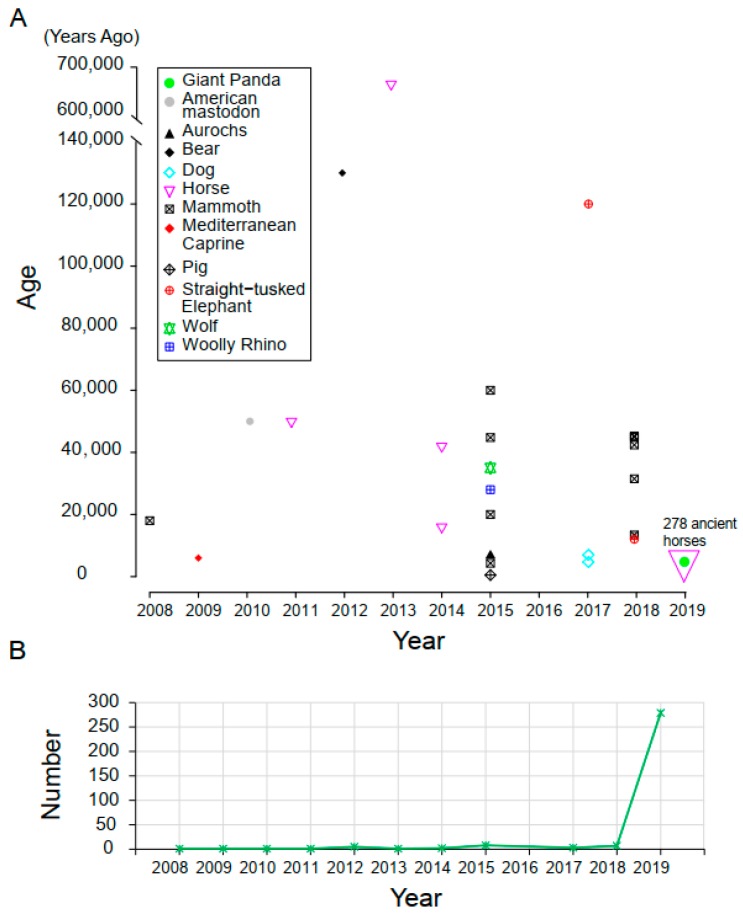
The investigation of whole genome sequenced ancient mammals from 2008 to 2019. (**A**) The species and age of whole genome sequenced ancient mammals from 2008 to 2019. (**B**) The number of whole genome sequenced ancient mammals from 2008 to 2019.

In this study, we used the whole-genome sequencing data of 12 ancient samples from five mammalian species to test different mapping algorithms, reference databases, mapping similarities, and query coverages, and searched for the best method and parameter combination that could improve ancient species identification. To include important data as much as we could to test guidelines we recommended in this study, we investigated the ancient mammal genomes whose whole genome have been sequenced (Figure 1). We then collected genome sequencing data of 152 ancient samples from four species and applied the recommended pipeline to these ancient samples. We also screened DNA fragments with deamination-induced changes (C-to-T and/or G-to-A) to improve the ability to identify genuine ancient DNA fragments and further improve species identification. The guidelines developed in this study demonstrated a significant improvement in species identification of ancient samples, which is otherwise challenged due to severe fragmentation and extremely low quantity of endogenous DNA.

## 2. Materials and Methods 

### 2.1. Samples and Data Resource

The whole-genome sequencing (WGS) data of twelve samples was used in this study, including six woolly mammoths (*Mammuthus primigenius*), three ancient humans (*Homo sapiens*) [25,26], one ancient horse [4], one ancient goat [27], and one ancient aurochs [28]. Over 200 woolly mammoth tusk samples were initially collected near the Lena River in Yakutia, Siberia, but the detailed geographic origin is unknown (Figure S1). We first tested the DNA extraction method, and DNA from 80 samples was successfully isolated. Of these 80 samples, six pieces of tusk (cut from the whole tusk) were selected based on their colors and different tusk diameters to ensure that they were not from the same individual. Besides, complete mitochondrial genomes of these six samples have been retrieved and assembled, five of them have been deposited to the CNSA (China National GeneBank Nucleotide Sequence Archive) (https://db.cngb.org/cnsa/) with accession number CNP0000277, demonstrating that they are from different individuals [29]. The six woolly mammoth samples were radiocarbon dated at the Beta Analytical Laboratory (Table 1). The WGS data of other samples were downloaded from NCBI, and their detailed information is listed in Table 1.

The raw sequencing data of six woolly mammoths have been deposited to the CNSA with accession number CNP0000209. The data has been simultaneously synchronized to EBI (https://www.ebi.ac.uk/) and NCBI (https://www.ncbi.nlm.nih.gov/) under the accession numbers PRJEB29510 and ERP111819, respectively. 

### 2.2. Sample Preparation and DNA Extraction 

About 1–3 mm of surface dirt was removed to decontaminate the tusk samples using sandpaper. We then soaked tusk samples in a 10–15% sodium hypochlorite solution for 10 min followed by cleaning using pure ethanol and high-performance liquid chromatography (HPLC)-grade water (this was done twice) for chemical decontamination. Prepared samples and extraction reagents were then put under UV light for 30 min before DNA extraction. Sample powder was obtained by drilling holes in the tusk samples using sterile drills at the low speed of 1000 rpm to protect the endogenous DNA from further degradation due to overheating. Approximately 200 mg of each sample material was used for DNA extraction by the silica-based method developed by Rohland and Hofreiter [30]. The high-sensitivity assay of the Qubit 3 Fluorometer (Invitrogen, Carlsbad, CA, USA) was used to measure the DNA concentration. All procedures were conducted in a standard ancient DNA laboratory. Sample decontamination and DNA extraction were carried out in two separate rooms. Two negative controls were performed during the DNA extraction to control possible contamination.

### 2.3. Library Preparation and Sequencing

We first constructed an Illumina-based DNA library using a start input DNA of ~10 ng from each tusk sample, as described in [31]. Ancient DNA is usually highly fragmented; we did not conduct any fragmentation procedures on DNA extracts before preparing the library. We then converted the library to one compatible with BGISEQ-500 sequencing platform (BGI, Shenzhen, China) by ligating BGISEQ adaptors, circularization, and rolling-cycle amplification to make DNA nano balls (DNBs). Finally, one BGISEQ-500 library was prepared for each sample and loaded on the BGISEQ-500 sequencer performing paired-end (PE) 100 sequencing. Three libraries were pooled to one pooling library. We sequenced two pooling libraries for six samples, generating ~2 billion reads in total. Negative control was performed during the ancient DNA library preparations to control for possible contaminations, especially from modern human DNA.

### 2.4. Processing of Sequencing Reads

We used AdapterRemoval [32] for trimming the sequencing adapters from raw reads, the Ns and low-quality bases at the ends of raw reads. We set a minimal number of three bases overlapping with the adapter sequences and a minimal base quality of 15. Reads with less than 30 bp after trimming were removed from the data set. We turned on the “collapse” mode of AdapterRemoval, because the DNA fragment in ancient DNA is usually very short, and PE 100 sequencing in our study could have led to some data redundancy. Some of the detailed parameters used are as follows: AdapterRemoval —qualitybase 33 —minadapteroverlap 3 —trimns —trimqualities —minquality 15 —collapse. 

### 2.5. Burrows-Wheeler Aligner Mapping and DNA Damage Analysis

We needed the DNA damage information—especially deamination characteristics of the DNA sequences—to design this species identification pipeline. So, the first step before constructing and testing this method was to detect the DNA damage on ancient DNA. BWA [33] (v0.7.10-r789) was used for mapping reads to reference genomes of each species (Table S1), allowing ≤2 differences in the seed. We used the “aln” function to map the FASTQ file to generate SAI files, with the seed disabled. All SAI files were then converted to SAM files using the BWA sample. We then used Picard Toolkit [34] to order the SAM files by chromosome and deduce the duplicated reads. SAM files were finally converted to BAM files using SAMtools [35] (v0.1.19-44428cd) for DNA damage analysis. All sequencing data generated in this study and those downloaded from NCBI were non-Uracil–DNA glycosylase (UDG)-treated. MapDamage 2.0 [36] was used to estimate the deamination-induced C-to-T changes at the ends of ancient DNA fragments with default parameters.

### 2.6. Exploring the Species Identification Pipeline

We first downloaded the nt database [37] from NCBI and extracted all the animal mitochondrial DNA (mtDNA) from the nt database to form an animal mtDNA database. Both of these databases were formatted by formatdb [38] (v2.2.26) before the BLAST search, with parameters “-p F -a F -o T -m 8”. Secondly, we used the BLASTn algorithm integrated into the BLASTall software [38] (v2.2.26) to map reads against the nt database and mtDNA database to compare the influence of different databases on species identification. To determine the species, we 1) sorted all valid mapping hits (VMH) (if one read was mapped at multiple places in one single sequence, we randomly selected one hit with the highest sequence similarity) against the database to obtain a species ranking (SR); 2) calculated the percentage of the VMH (PoVMH) for each item in the SR; 3) compared the PoVMH of the top one species and the second-ranked one to determine the most likely species. We also tested different similarities and different query coverages to improve the identification accuracy. The FASTQ data of the N1 sample were divided into 37 groups by reads number, ranging from 100 to 5,000,000 in order to evaluate the minimal reads number required to obtain a stable SR before conducting the BLAST search against the nt database. This was done because performing a BLAST search using the nt database is tedious and time-consuming. Thirdly, we used the BWA function “mem”, BWA function “aln”, and BLAST search to map the reads of all samples against the best database (identified through the second step), and compared the different mapping algorithms. Fourthly, we applied the best method and parameters summarized from the above procedures to 152 ancient samples with one million randomly selected reads. Finally, we screened reads from the mapping results according to the C-to-T and/or G-to-A changes at the ends of ancient DNA fragments to improve the accuracy of species identification. Here, we used the parameter “-m 8” for BLAST search to output the detailed sequence differences. To increase the convenience of ancient species identification, we also wrote a software script with Perl, AncSid. This software with a detailed manual has been submitted here: https://github.com/tianminglan/AncSid. 

## 3. Results

### 3.1. Samples and Data Description

The age of the samples used for exploring the guidelines of ancient species identification ranged from several hundred years before present (BP) to an extreme of ~700 k years BP. The average length of endogenous DNA fragments recovered from these samples was not longer than 73 bp. The proportion of endogenous DNA varied among the samples, of which the endogenous DNA of the ~700 k years BP ancient horse was extremely low (~0.43%), see Table 1 [4]. In order to further test the application of this guideline and determine whether it can be successfully used in BGISEQ-500 sequencing data, we performed the whole-genome sequencing of six woolly mammoths from tusk samples. The average length of DNA fragments was not longer than 100 bp, and the percentage of endogenous DNA ranged from 0.54% to 59.51% (Table 1). We could not detect DNA in all negative controls by the Qubit high-sensitivity assay, indicating that no contamination was induced during the DNA extraction and library preparation. However, it is still possible that the woolly mammoth samples were contaminated with modern human DNA during unearthing, collection, and transportation of the samples. We further mapped reads to the human reference genome (GRCh38), and only 0.05% reads were found to originate from a modern human, which was filtered through mapping against the elephant genome. All the data obtained had an obvious DNA damage pattern (Figure S2). The frequencies of C-to-T and/or G-to-A changes at the ends of DNA fragments varied widely among different samples, ranging from ~2% to ~80%.

### 3.2. Basic Local Alignment Search Tool Search Using Nucleotide (nt) and Mitochondrial DNA (mtDNA) Databases

We found that the SR of the first five species never changed when the reads number reached 20,000 in the BLAST search. To ensure the reliability, we ran the BLAST search against the nt database using 100,000 reads per sample. Using this criterion, we simultaneously performed the BLAST search against the nt database and the animal mtDNA database using the same FASTA data of all samples. For the three human samples, both databases gave the correct identification results, with the top one species being the SR *Homo sapiens*. The PoVMH for the top one species was significantly higher than that of the second-ranked species (*Z* = -5.125, *P* = 2.20 × 10^-10^). However, for non-human mammal samples, only the ancient cattle sample was successfully identified using the nt database, while the other eight samples including ancient goat, ancient horse, and woolly mammoth could not be identified (Table S2). On the contrary, we successfully identified eight of the nine non-human mammal samples using the animal mtDNA database (Table 2, Table S2). Although the ancient goat samples were identified as *Bos taurus*, *Capra hircus* occupied the second place at all similarity levels (*L*s). To further confirm the effectiveness of the mtDNA database, we performed the BLAST search using the full sequence data listed in Table 1. All samples were successfully identified when we selected VMH with similarities higher than 94%, 96%, and 98%. These results showed that the animal mtDNA database was more suitable for ancient species identification than the whole nt database. To further lower the database bias, we selected only one complete or nearly complete mitochondrial genome sequence for each mammal species in order to form a new database to be used for testing BLAST-search-based species identification. However, only eight samples were successfully identified with a similarity ≥ 98%, including three human samples, an ancient goat sample, and four woolly mammoth samples; the accuracy was 66.67% (Table S3). Taking the computation time into account, the BLAST search using the nt database is time-consuming. Thus, the whole mtDNA database should be the first choice for the species identification of ancient mammals.

### 3.3. Screening the Mapping Results by Sequence Similarity

**Figure 2:**
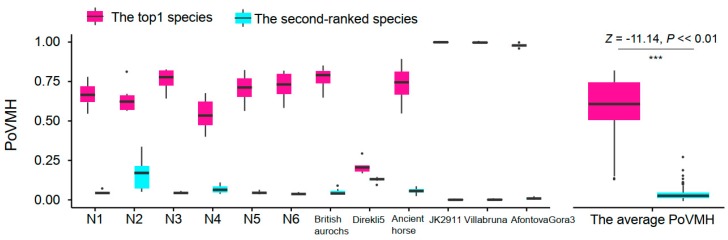
The percentage of valid mapping hits (PoVMH) of the top one and the second-ranked species in the species ranking (SR) based on the BLAST search results generated using the whole mtDNA database. The range is represented by whiskers, individual data points are shown using dots, the upper quartiles and lower quartiles are denoted by the boxes, and the medians are shown using the central lines. The *n* in each species was seven, and the *n* in the average PoVMH was 84.

We used the whole mtDNA database to evaluate the influence of sequence similarity and query coverage on identification accuracy. Because this database is much smaller than the nt database, all clean reads of each sample listed in Table 1 were used to perform the BLAST search. For sequence similarity, we divided the test into six levels (*L* ≥ 90%, *L* ≥ 92%, *L* ≥ 94%, *L* ≥ 96%, *L* ≥ 98%, *L* = 100%). The top one species in the SR gave correct identification results for all 12 samples when *L* reached 94%, and the PoVMH of the top one species was significantly higher than the second-ranked species (Figure 2). 

**Figure 3:**
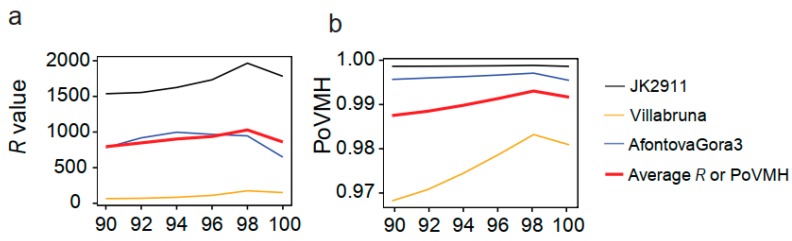

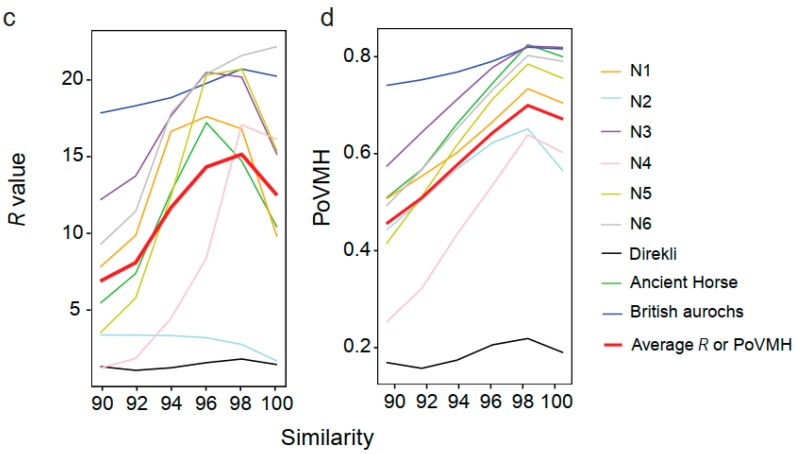
The *R* value and PoVMH of the top one species under different similarities based on the BLAST search results generated using whole mtDNA database. 90: 90 ≤ *L* ≤ 100; 92: 92 ≤ *L* ≤ 100; 94: 94 ≤ *L* ≤ 100; 96: 96 ≤ *L* ≤ 100; 98: 98 ≤ *L* ≤ 100; 100: *L* = 100. (**a**) The *R* values under different similarities for human samples; (**b**) the PoVMH under different similarities for human samples; (**c**) the *R* values under different similarities for mammal samples; (**d**) the PoVMH under different similarities for mammal samples.

Only the ancient goat could not be accurately identified when *L* ≥ 90% or *L* ≥ 92% (Table S4). We further compared the ratio of the PoVMH (*R*) between the top 1 species and the second-ranked species to quantify the differences among *L*s. In human samples, the PoVMH for the top 1 species was higher than 96%, and was lower than 1.5% for the second-ranked species in all *L*s. No significant difference was found for *R*s among *L*s (*df* = 5, *F* = 0.22, *P* = 1). However, for non-human samples, the PoVMH number of the top one species and the *R*s presented an upward trend from 90% to 98%, but decreased when *L* reached 100% (Figure 3), which was more obvious than in human samples (*df* = 5, *F* = 2.1, *P* = 0.082). Although *R* in *L* ≥ 98% was significantly higher than that in *L* ≥ 90% (*P* = 0.014) and *L* ≥ 92% (*P* = 0.034), no difference was found among *L*s of 100%, 98%, 96%, and 94% (Table S5). Because deamination is a common characteristic of ancient DNA, we expected the differences between the query sequence and the database sequence. We then re-evaluated the five levels (*L* ≥ 90% and *L* < 100%, *L* ≥ 92% and *L* < 100%, *L* ≥ 94% and *L* < 100%, *L* ≥ 96% and *L* < 100%, *L* ≥ 98% and *L* < 100%) by eliminating VMH with 100% similarity in each *L* group. No significant improvement was found in *R*s when compared to *L*s with 100% similarity (Z = -1.056, *P* = 0.291), but the *R* in *L* ≥ 98% was significantly higher than all other *Ls* (Table S6), which showed the advantage of the similarity threshold of 98%.

### 3.4. Screening the Mapping Results by Query Coverages

The local alignment performed by BLAST search led to partial alignments of the query. In order to evaluate the influence of query coverage in species identification, we compared the identification results under different query coverages (*C*, *C* < 90; 90 ≤ *C* < 92; 92 ≤ *C* < 94; 94 ≤ *C* < 96; 96 ≤ *C* < 98; 98 ≤ *C* < 100; 98 ≤ *C* ≤ 100, and *C* = 100). A 100% accuracy was obtained for all human samples even when the query coverage was less than 90% (Table S7), which showed that the query coverage did not influence the ancient human species identification. However, for ancient mammal samples, the accuracy decreased to 83.33%. All incorrectly identified cases were concentrated on query coverage of less than 96%. The accuracy was stable at 100% when query coverage was higher than 96%. In addition, query coverage did not influence the *R*s (*df* = 6, *F* = 1.099, *P* = 0.378), but the VMH of the top one species for the 100% query coverage were significantly higher than those for the query coverage of less than 100% (Table S8).

### 3.5. Mapping Using the BWA Functions “aln” and “mem”.

BLAST performs local alignment and matches within the query, which may lose the C-to-T and G-to-A changes at ends of the ancient DNA fragments. Therefore, to keep more deamination information to help in screening the true endogenous DNA and further improve the accuracy of species identification, we carried out the BWA mapping against the whole animal mtDNA database using both “aln” and “mem” functions. We calculated the *R*s of all *L* groups mentioned in Section 3.3 for all samples. Both BWA “aln” and BWA “mem” misidentified the ancient cattle, the ancient horse and one woolly mammoth sample (N2) as *Chrysomya albiceps*, *Andalucia godoyi*, and *Stenopirates sp.*, respectively. No remarkable difference was found in *R*s between BWA *aln* and *mem* algorithms for the nine successfully identified samples (145 ± 316, 165 ± 341, *Z* = -0.694, *P* = 0.488). However, the *R*s in the BLAST search results were significantly higher than BWA “aln” (*Z* = -3.309, *P* = 0.001) and “mem” (*Z* = -2.624, *P* = 0.009) methods. Besides, the VMH of the top one species in the BLAST search was also remarkably higher than BWA *“aln”* (*Z* = -11.983, *P* = 4.36 x 10^-33^) and “*mem*” (*Z* = -12.175, *P* = 4.25 x 10^-34^) methods.

### 3.6. Testing the Recommended Method and Parameters for Ancient Mammal Species Identification

We collected the whole-genome sequencing data from a 5000-year-old panda [39], one sixteenth century pig [40], and 149 ancient horse samples [41]. We performed the species identification using BLAST search with the mtDNA database. We further used the similarity parameter of *L* ≥ 98% and *L* < 100% and the query coverage parameter of C ≥ 96% to filter the mapping results. Finally, 150 (98.68%) ancient samples were successfully identified (Table S9). However, two ancient horse samples could not be identified. Further analysis of these two ancient horse samples showed that only one and two reads were actually mapped to the mtDNA database for the two samples, respectively. We inferred that the failure in identification of these two samples could be the result of an extremely low content of the endogenous DNA. Of these 152 samples, 150 libraries were UDG treated, we did not perform the further filtration using deamination characteristics.

### 3.7. Screening the Mapping Results Using Deamination Characteristics 

**Figure 4:**
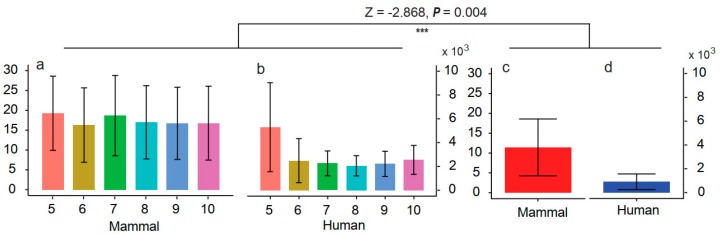
The comparison of *R* values before and after screening the reads based on deamination-induced C-to-T and/or G-to-A change at ends of DNA fragments. The numbers 5 to 10 on the *x*-axis denote the first and last *X* bases for screening the reads with C-to-T and/or G-to-A changes. (**a**) and (**b**) show the average *R* values of 12 samples after screening, and (**c**) and (**d**) show the average *R* values of 12 samples before the deamination-based screening. The BLAST search and whole mtDNA database were used in this comparison. The error bars denote the standard error.

As mentioned above, deamination is one of the most important characteristics of ancient DNA, which can be of help when selecting the true ancient DNA fragments to improve the accuracy of species identification. We screened reads with at least one C-to-T or G-to-A change at the ends of ancient DNA according to the damage pattern of each sample. The whole animal mtDNA database and similarity of ≥98% were selected to evaluate their effectiveness, because this combination presents better identification accuracy. We divided this test into six groups by setting six thresholds: screening DNA fragments with at least one C-to-T or G-to-A change within the first or last 5/6/7/8/9/10 bases were selected. All the samples except the ancient horse were accurately identified in all six groups (Table S10). For the ancient horse sample, we could not obtain the correct identification when the threshold was set to 5, 6 or 7, but *Equus caballus* appeared at the top one position when the threshold ranged from 8 to 10. No significant differences were found in *R*s among these six groups (*df* = 5, *F* = 0.979, *P* = 0.437). However, these *R*s were significantly higher than those of samples without deamination screening (*Z* = -2.868, *P* = 0.004) (Figure 4).

## 4. Discussion

DNA in very old samples is often severely fragmented and highly contaminated, and thus it is very difficult to successfully amplify for species identification—even for very short amplicons (e.g. <100 bp) [14,16,21,22]. In another study, 80 woolly mammoth samples were amplified using a species-specific primer pair with an amplicon size of 114 bp, and 19 samples could not be successfully sequenced. In this study, the average length of DNA fragments was less than 100 bp, and was even shorter than 50 bp for some samples. Although the maximum length of the DNA fragments from woolly mammoth samples can reach up to 500 bp, the total proportion of DNA fragments larger than 150 bp was less than 5%, and only one sequence was found to support an amplification of 500 bp. The maximum ancient DNA length was only 73 bp for the ancient cattle sample, and was 93 bp for the ancient horse sample. These short DNA fragment sizes are almost impossible to achieve using PCR amplifications, thus presenting a challenge for ancient species identification. However, the guidelines we have demonstrated here fill this gap and allowed the successful identification of 162 (98.78%) ancient samples, showing their advantages in ancient species identification. 

### 4.1. Comparing the nt and mtDNA Databases to Improve the Identification Accuracy

The identification accuracy was only 33.33% after performing BLAST search of the nt database. This rate decreased to 11.11% when we removed the human samples. However, all samples were successfully identified by changing the database to the whole animal mtDNA database. The zero failure rate was obviously lower than other studies based on PCR amplification plus BLAST search [21,42], demonstrating the superiority of the mtDNA database for the species identification of ancient mammals. 

With the plummeting sequencing costs, the whole-genome sequencing of approximately 4690 eukaryote species/subspecies has been completed, including 1359 animals and 427 plants [43]. However, this is a very small proportion considering a global estimate of 8.7 million eukaryotic species [44]. As a result, most of the sequence data in GenBank are highly biased to a few species, especially to human, with thousands of whole genomes deposited in the database [45]. This enabled us to obtain 100% identification accuracy for human samples in both the nt and mtDNA databases based on BLAST search. However, only one ancient mammal sample (the ancient cattle) was successfully identified, and the accuracy decreased by nine times when compared to that of human samples. We inferred that this might have resulted due to the biased data resource, because shorter DNA fragments leads to the occurrence of more random wrong hits. 

The mitochondrial genome is much smaller and simpler than the nuclear genome, and it is valuable in many research fields. This has rapidly increased the number of mitochondrial genome sequences deposited in GenBank, making it the most-sequenced type of eukaryotic chromosome [46]. The 100% accuracy for ancient mammal species identification showed that the data bias in the animal mtDNA database was definitely lower than the whole nt database, although this bias would always exist. To mitigate this bias, we selected only one complete or near-complete mitochondrial genome for each animal species to form a new database, but the accuracy was only 66.67%. This indicated that a single mitochondrial genome for each species cannot represent the overall intraspecies genetic diversity, especially for ancient animals that might have a higher genetic distance than that seen in modern species, which might be even higher considering the deamination-induced C-to-T changes [47]. In this study, the whole animal mtDNA database presented enough power to determine the species of origin of ancient mammal samples. However, more reasonable sample sizes should be estimated to form a reference database in the future [48]. 

### 4.2. Evaluating the Influence of Sequencing Similarity and Coverage on Identification Accuracy

For modern DNA, a relatively higher similarity (e.g. 98–100%) is usually selected to identify the species [49], because the genetic distance of mtDNA is less than 2% in many species [48]. Although the 100% match between query sequence and reference sequence can be a good criterion in species identification [42], the *R*s in our study decreased when the *L* reached 100% (Figure 3), even lower than *R*s when *L* was ≥94% in some cases, indicating that the criterion of 100% match is not the best choice in the BLAST search of ancient mammals. The age of ancient samples can often be dated back to several thousand or even hundreds of thousands of years [3,4,27,50]. The serious shortage of ancient DNA sequences in public databases makes it difficult to perform comparisons within ancient samples. Under this situation, we have to subject the query sequences to BLAST against the corresponding modern individuals, which could further increase the pairwise difference within species. This difference might be even more pronounced when considering the DNA damage in ancient DNA fragments. Due to the reasons above, a relatively lower similarity (e.g. ≥98%) rather than 100% is suggested in the BLAST-based species identification of ancient mammals. 

BLASTall is a local alignment tool which performs matches within the query sequences. This may suggest that higher similarity does not always represent better results, when considering the coverage of the mapped DNA fragment. Although we used the same similarity of ≥98% for testing the influence of query coverage in species identification, lower query coverage presented the lower identification accuracy (Table 2). In our study, all incorrectly identified cases were found to have a query coverage of less than 96%. This might have been caused by very short ancient DNA fragments, as lower query coverage makes the sequence shorter, resulting in increased random hits. We therefore suggest the selection of a query coverage higher than 96% to improve the accuracy in the identification of ancient mammals, although this may not be suitable for long sequences. We further tested the BLAST search with the recommended similarity parameter of *L* ≥ 98% and *L* < 100% and the query coverage parameter of C ≥ 96% using 152 more ancient samples; 98.68% of the samples were successfully identified, supporting the accuracy of our guidelines.

### 4.3. Comparison of Different Mapping Algorithms to Improve Identification Accuracy

In contrast to the BLAST search, the BWA algorithm maps the entire query sequences to the reference database without any trimming. Normally, BWA “aln” or “mem” algorithms are both expected to retain more deamination information to help in the screening of true ancient DNA fragments. However, the accuracy of BWA “aln” and BWA “mem” were both 75%, which was lower than the BLAST search. We inferred that the semi-global alignment implanted in BWA resulted in too many failed matches, because deamination frequently occurs at the ends of ancient DNA fragments [51]. The low significance level of the VMH of the top one species supported our hypothesis. We did not perform further BWA mapping with a larger data set to meet the same VMH as BLAST search; instead, we used BLAST search to determine the species of ancient mammals when the sequence data were limited. However, if we could obtain the same level of VMH as BLAST search, BWA mapping results could provide more deamination information.

### 4.4. Improving Identification Accuracy by Using Deamination Characteristics

**Figure 5:**
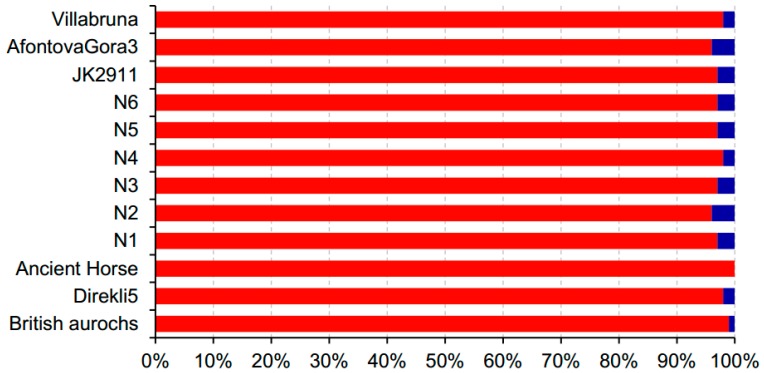
The comparison of VMH before and after screening the reads based on deamination-induced C-to-T and/or G-to-A changes at the ends of DNA fragments. The red area in the bars represents the proportion of VMH before the screening and the blue area shows the proportion after the screening. The BLAST search and whole mtDNA database were used in this comparison.

The deamination information is very important when selecting endogenous DNA—especially in circumstances where the rate of contamination with modern DNA cannot be accurately evaluated [51]. We expected to significantly increase the proportion of endogenous DNA by screening reads with the C-to-T and/or G-to-A changes at the overhangs [52,53,54]. In this study, we screened the DNA sequences with C-to-T and/or G-to-A mutations at the termini. We could not obtain global alignment because of the local alignment algorithm of the BLAST search. Therefore, the BLAST search may automatically trim bases found at the termini of the query in the event of poor sequence matches. This might have resulted in the absence of mutations at the first or last four bases in our study. However, from the first or last fifth to tenth positions, C-to-T and/or G-to-A changes were observed. Gathering the reads with potential deamination-induced mutations significantly increased the *R*s. For Ice_Villabruna, all the sequences obtained were from *Homo sapiens*, showing the strong advantage for improving the accuracy of ancient species identification. It is noteworthy that the VMH sharply decreased when the screening of C-to-T and/or G-to-A mutations was performed at the overhangs (Figure 5). We therefore suggest screening within the first or last 10 bases to increase VMH, although no significant difference was found for *R*s among the thresholds used to select targeting reads.

### 4.5. Influence of the Sample Age, Fragment Length, Proportion of Endogenous DNA, and Sequencing Platform on the Identification Accuracy

Woolly mammoth samples in this study were sequenced using the BGISEQ-500 sequencer. The additional sequence data downloaded from GenBank was generated by Illumina series (Table 1), which is presently the dominant sequencing platform. Mak et al. [55] extensively compared the BGISEQ-500 and Illumina series for paleogenomic sequencing, and suggested that the BGISEQ-500 was comparable to Illumina series and it presents a valid platform for generating paleogenomic data. In this study, the degree of accuracy for woolly mammoth samples was high and stable under various conditions (Table 2). Nevertheless, the *R*s were not significantly higher than other mammals (Z = -1.086, *P* = 0.277, excluding human samples) when we assessed the results generated by BLAST search against the whole mtDNA database. As such, we cannot conclusively determine which platform performed better considering the differences in data size, age, and the proportion of endogenous DNA. However, the BGISEQ-500 platform presented a highly effective ancient species identification for woolly mammoth, which further supports that the BGISEQ-500 is a potential alternative platform for ancient DNA generation.

The preservation conditions to which the ancient samples should be subjected are very complicated, including temperature, minerals, humidity, microorganisms, salinity, and so on. The age of samples might not always be of close relevance to DNA fragmentation and the proportion of endogenous DNA [19]. Although the ancient horse sample was the oldest sample with an extreme age of ca. 560–780 k years (i.e., 70 times older than the ancient goat sample), the *R*s of the ancient horse were surprisingly higher than those of the ancient goat sample (*Z* = -2.882, *P* = 0.004) when we performed the BLAST search against the whole mtDNA database. Thus, the age of samples might not be a stable indicator for evaluating the difficulties in ancient species identification using NGS technology and BLAST search. Similarly, we did not find a strong correlation between the identification accuracy and the length of the DNA fragment, nor the proportion of endogenous DNA in this study. The proportion of endogenous DNA from N1 was nearly 110 times higher than N5, but the *R*s for N1 were not significantly higher than those of N5 (*Z* = 0, *P* = 1). It was therefore difficult to evaluate how a single aspect could influence the species identification of ancient mammals. DNA damage tends to increase over time [19], which enabled us to find true ancient DNA sequences. The damage-induced C-to-T changes observed in our study significantly increased the *R*s when we screened the reads with this characteristic. However, a relatively larger data set is needed to screen the damaged ancient DNA fragments in order to meet the requirement of the minimal reads number.

## 5. Conclusions

In this study, we successfully identified the species of different ancient mammalian samples, representing some extremely old samples with heavily degraded and contaminated DNA. By optimizing the mapping procedure and filtering parameters, we were able to successfully perform the screening and identification of the intended ancient DNA. Ancient human samples demonstrated better tolerance to different conditions, and they could always be successfully identified—possibly due to the huge amount of human genome sequences in the database. For ancient mammal samples, we present several suggestions to create guidelines for the identification of species from ancient samples: 1) the complete mtDNA database in conjunction with BLAST search should be the first choice for the species identification of ancient mammals; 2) BLAST search can be preferably used to map against the reference database, although the BWA algorithm will retain more deamination information in mapping results; 3) a similarity of ≥98% rather than 100% is preferable when performing the BLAST search; 4) query coverage higher than 96% provides a better accuracy for the species identification of ancient mammals; 5) screening DNA fragments with C-to-T and/or G-to-A within the first and last 10 bases provides better *Rs* while keeping more VMH. Finally, we did not find an obvious relationship between the identification accuracy and the sample age, DNA fragment length, or the proportion of endogenous DNA, due to the extremely complicated preservation conditions among ancient remnants, which in contrast demonstrated the universal property of this guideline. 

## Figures and Tables

**Table 1:** The description of the next-generation sequencing (NGS) data and samples.

Species	Sample ID	Age (kyr BP)	Data Sources	Sequencing Platform	Reads Number	Bases Number	Average Length (bp)	Proportion of Endogenous DNA
*Mammuthus primigenius*	N1	26	Sequencing	BGISEQ-500	1.00 x 10^7^	9.32 x 10^8^	93.17	59.51%
*Mammuthus primigenius*	N2	28	Sequencing	BGISEQ-500	1.00 x 10^7^	8.76 x 10^8^	87.57	0.93%
*Mammuthus primigenius*	N3	>43.5	Sequencing	BGISEQ-500	1.00 x 10^7^	8.70 x 10^8^	87.03	34.50%
*Mammuthus primigenius*	N6	>43.5	Sequencing	BGISEQ-500	1.00 x 10^7^	9.07 x 10^8^	90.69	1.51%
*Mammuthus primigenius*	N9	>43.5	Sequencing	BGISEQ-500	1.00 x 10^7^	8.99 x 10^8^	89.87	0.54%
*Mammuthus primigenius*	N12	17	Sequencing	BGISEQ-500	1.00 x 10^7^	9.10 x 10^8^	91.02	21.90%
*Homo sapiens*	JK2911	2.7	Schuenemann et al.	Illumina HiSeq 2500	9.74 x 10^5^	7.08 x 10^7^	72.62	39.20%
*Homo sapiens*	AfontovaGora3	17	Fu et al.	Illumina HiSeq 2500	8.88 x 10^5^	5.17 x 10^7^	58.18	44.64%
*Homo sapiens*	Villabruna	14	Fu et al.	Illumina HiSeq 2500	1.22 x 10^7^	6.69 x 10^7^	55.02	41.13%
*Capra aegagrus hircus*	Direkli5	11.5	Daly et al.	Illumina HiSeq 2000	3.04 x 10^7^	1.40 x 10^7^	45.94	5.29%
*Bos primigenius*	British aurochs	6.7	Park et al.	Illumina Genome Analyzer IIx	7.51 x 10^7^	3.48 x 10^9^	46.29	5.91%
Ancient horse	Ancient horse	560-780	Orlando et al.	Illumina HiSeq 2000	6.27 x 10^6^	3.34 x 10^8^	53.23	0.43%

BP: before present.

**Table 2:** Success rate of ancient species identification under different conditions.

Conditions		nt Database	mtDNA Database			
			Animal mtDNA Database (Whole)			Animal mtDNA Database (Partial)
		BLASTall	BLASTall	BWA aln	BWA mem	BLASTall
Similarity levels (*L*, %)	90 ≤ *L* ≤ 100	4/12	11/12	9/12	9/12	5/12
	92 ≤ *L* ≤ 100	4/12	11/12	9/12	9/12	5/12
	94 ≤ *L* ≤ 100	4/12	12/12	9/12	9/12	6/12
	96 ≤ *L* ≤ 100	4/12	12/12	9/12	9/12	6/12
	98 ≤ *L* ≤ 100	4/12	12/12	9/12	9/12	8/12
	*L* = 100	4/12	12/12	9/12	9/12	5/12
	90 ≤ *L* < 100	4/12	10/12	7/12	7/12	3/12
	92 ≤ *L* < 100	4/12	11/12	7/12	7/12	3/12
	94 ≤ *L* < 100	4/12	12/12	8/12	9/12	4/12
	96 ≤ *L* < 100	4/12	12/12	9/12	9/12	5/12
	98 ≤ *L* < 100	4/12	12/12	10/12	10/12	8/12
Query coverage (*C*, %)	*C* < 90	--	7/12	--	--	--
	*C* ≥ 90-92	--	7/12	--	--	--
	*C* ≥ 92-94	--	11/12	--	--	--
	*C* ≥ 94-96	--	11/12	--	--	--
	*C* ≥ 96-98	--	12/12	--	--	--
	*C* ≥ 98-100	--	12/12	--	--	--
	*C* = 100	--	12/12	--	--	--
The first and last *X* bases for screening reads with C-to-T and/or G-to-A changes	*X* = 5	--	11/12	--	--	--
	*X* = 6	--	11/12	--	--	--
	*X* = 7	--	11/12	--	--	--
	*X* = 8	--	12/12	--	--	--
	*X* = 9	--	12/12	--	--	--

We used BLASTall with the similarity of ≥98% to test the query coverage and deamination screening. BWA: Burrows-Wheeler aligner; mtDNA: Mitochondrial DNA; nt: Nucleotide; aln, mem: BWA functions

## Supplementary Materials

The following are available online at https://www.mdpi.com/2073-4425/10/7/509/s1, Table S1: The reference genome used in BWA mapping, Table S2: Comparison of nt database and mtDNA database for ancient species identification based on BLAST search, Table S3: Results of BLAST search generated using animal mtDNA database with selected 3220 sequences, Table S4: Results of BLAST search generated using whole animal mtDNA database; Table S5: Analysis of One-Way ANOVA with LSD for R values of different similarities by use of whole animal mtDNA database based on BLAST search, Table S6: Analysis of One-Way ANOVA with LSD for *R* values of different similarities (without 100%) by use of whole animal mtDNA database based on BLAST search; Table S7: Comparisons of species identification under different query coverages by use of BLAST search based on whole animal mtDNA database; Table S8: Analysis of One-Way ANOVA with LSD for VMH of different query coverage by use of whole animal mtDNA database based on BLAST search; Table S9: Testing of the best method and parameters for ancient mammal species identification using 152 ancient samples; Table S10: Comparisons of species identification under different screening conditions based on deamination induced C-to-T and/or G-to-A changes; Figure S1: The sampling area of woolly mammoth samples. The dark circle shows the sampling area; Figure S2: Patterns of cytosine deamination in DNA libraries of 12; samples. The y axis shows the frequencies of nucleotide substitutions, and the x axis shows the distance from 5’ and 3’ ends. Red: C-to-T. Blue: G-to-A.
