# Supplementary material for: Improving Species Identification of Ancient Mammals Based on Next-Generation Sequencing Data
Source: Genes (Basel). 2019 Jul 5;10(7):509. doi: 10.3390/genes10070509 (PMC6679096; doi:10.3390/genes10070509)
Supplement: Supplementary file 1 [file genes-10-00509-s001.pdf]

Improving Species Identification of Ancient Mammals Based on Next-Generation Sequencing Data

Tian Ming Lan †, Yu Lin †, Jacob Njaramba-Ngatia, Xiao Sen Guo, Ren Gui Li, Hai Meng Li, Sunil Kumar-Sahu, Xie Wang, Xiu Juan Yang, Hua Bing Guo, Wen Hao Xu, Karsten Kristiansen, Huan Liu *, Yan Chun Xu *

Table S1. The reference genome used in BWA mapping

Species	No. of Reference genome	Version	Website
Bos primigenius	bosTau8	Jun. 2014	http://hgdownload.cse.ucsc.edu/goldenPath/bosTau8/bigZips/bosTau8.fa.gz
Ancient horse	equCab2	Sep. 2007	http://hgdownload.cse.ucsc.edu/goldenPath/equCab2/bigZips/chromFa.tar.gz
Capra aegagrus hircus	GCF_001704415.1_ARS1	Aug. 2016	ftp://ftp.ncbi.nlm.nih.gov/genomes/all/GCF/001/704/415/GCF_001704415.1_ARS1/GCF_001704415.1_ARS1_genomic.fna.gz
Homo sapiens	GRCh38	Dec. 2013	http://hgdownload.cse.ucsc.edu/goldenPath/hg38/bigZips/hg38.fa.gz
Mammuthus primigenius	loxAfr4	May. 2014	ftp://ftp.broadinstitute.org/distribution/assemblies/mammals/elephant/loxAfr4/Chromosomes.v2.fasta.gz

‡ The mitochondrial genome in the reference genome of woolly mammoth was replaced by a another mitogenome (Acc No. DQ188829.2)

Table S2. Comparision of nt database and mtDNA database for ancient species identification based on BLAST search

Samples	Similarity Ls (%)	nt database (100K)				mtDNA database (100K)			
		SR	VMH	PoVMH (%)	***R***	SR	VMH	PoVMH (%)	***R***
British aurochs	L=100	*Bos taurus*	241,218	24.18	2.50	*Bos taurus*	878	90.89	23.73
		Uncultured bacterium	96,608	9.69		*Bos indicus*	37	3.83	
	90≤L≤100	*Bos taurus*	864,958	33.87	3.73	*Bos taurus*	1,122	85.58	22.90
		Ovis aries	231,944	9.08		*Bos indicus*	49	3.74	
	92≤L≤100	*Bos taurus*	829,441	33.74	3.84	*Bos taurus*	1,122	85.71	22.90
		Ovis aries	216,002	8.79		*Bos indicus*	49	3.74	
	94≤L≤100	*Bos taurus*	730,751	33.10	3.80	*Bos taurus*	1,118	87.41	23.79
		Uncultured bacterium	192,331	8.71		*Bos indicus*	47	3.67	
	96≤L≤100	*Bos taurus*	552,385	31.87	3.64	*Bos taurus*	1,106	89.63	25.14
		Uncultured bacterium	151,724	8.75		*Bos indicus*	44	3.57	
	98≤L≤100	*Bos taurus*	290,131	26.33	2.73	*Bos taurus*	878	90.89	23.73
		Uncultured bacterium	106,159	9.63		*Bos indicus*	37	3.83	
JK2911	L=100	*Homo sapiens*	850,861,037	99.03	996.48	*Homo sapiens*	5,794,791	99.74	703.17
		Pan troglodytes	853,864	0.10		*Pan troglodytes*	8,241	0.14	
	90≤L≤100	*Homo sapiens*	1,356,437,830	94.90	253.72	*Homo sapiens*	8,879,276	99.72	582.78
		Pan troglodytes	5,346,180	0.37		*Pan troglodytes*	15,236	0.17	
	92≤L≤100	*Homo sapiens*	1,356,194,998	95.95	270.79	*Homo sapiens*	8,865,706	99.73	587.29
		Pan troglodytes	5,008,295	0.35		*Pan troglodytes*	15,096	0.17	

	94≤L≤100	*Homo sapiens*	1,355,273,046	96.96	322.92	*Homo sapiens*	8,833,784	99.74	614.65
		Pan troglodytes	4,196,937	0.30		*Pan troglodytes*	14,372	0.16	
	96≤L≤100	*Homo sapiens*	1,333,940,447	98.05	450.99	*Homo sapiens*	8,665,518	99.75	666.94
		Pan troglodytes	2,957,827	0.22		*Pan troglodytes*	12,993	0.15	
	98≤L≤100	*Homo sapiens*	1,185,600,000	98.94	772.77	*Homo sapiens*	7,774,738	99.77	743.14
		Pan troglodytes	1,534,221	0.13		*Pan troglodytes*	10,462	0.13	
Direkli5	L=100	Uncultured bacterium	212,162	14.02	1.37	*Bos taurus*	242	23.45	1.85
		Bos taurus	155,355	10.27		*Capra hircus*	131	12.69	
	90≤L≤100	*Bos taurus*	783,580	20.25	2.26	*Bos taurus*	511	16.27	1.17
		Uncultured bacterium	346,913	8.96		*Capra hircus*	435	13.85	
	92≤L≤100	*Bos taurus*	721,909	20.36	2.15	*Bos taurus*	507	18.26	1.23
		Uncultured bacterium	335,757	9.47		*Capra hircus*	413	14.88	
	94≤L≤100	*Bos taurus*	608,924	19.51	1.90	*Bos taurus*	417	18.01	1.72
		Uncultured bacterium	320,817	10.28		*Capra hircus*	242	10.45	
	96≤L≤100	*Bos taurus*	420,717	16.82	1.49	*Bos taurus*	402	20.04	1.76
		Uncultured bacterium	282,921	11.31		*Capra hircus*	229	11.42	
	98≤L≤100	Uncultured bacterium	224,753	13.42	1.14	*Bos taurus*	325	19.67	1.53
		Bos taurus	197,470	11.79		*Capra hircus*	213	12.89	
Ancient horse	L=100	Uncultured bacterium	177,104	17.34	8.62	*Equus caballus*	160	91.95	26.67
		Mus musculus	20,548	2.01		*Equus przewalskii*	6	3.45	

	90≤L≤100	Uncultured bacterium	643,460	17.73	13.33	*Equus caballus*	160	42.44	2.32
		Cyprinus carpio	48,262	1.33		*Reclinomonas americana*	69	18.30	
	92≤L≤100	Uncultured bacterium	626,106	18.46	13.85	*Equus caballus*	160	50.31	4.00
		Homo sapiens	45,193	1.33		*Reclinomonas americana*	40	12.58	
	94≤L≤100	Uncultured bacterium	551,008	19.05	13.99	*Equus caballus*	160	58.61	5.93
		Homo sapiens	39,375	1.36		*Reclinomonas americana*	27	9.89	
	96≤L≤100	Uncultured bacterium	429,150	19.42	14.03	*Equus caballus*	160	74.42	10.67
		Homo sapiens	30,581	1.38		*Andalucia godoyi*	15	6.98	
	98≤L≤100	Uncultured bacterium	239,897	17.83	10.73	*Equus caballus*	160	90.40	26.67
		Mus musculus	22,366	1.66		*Equus przewalskii*	6	3.39	
AfontovaGora3	L=100	*Homo sapiens*	35,106,738	66.31	8.22	*Homo sapiens*	131,479	99.40	369.32
		Pan troglodytes	4,268,849	8.06		*Pan troglodytes*	356	0.27	
	90≤L≤100	*Homo sapiens*	114,385,005	64.38	7.62	*Homo sapiens*	350,591	99.48	596.24
		Pan troglodytes	15,005,101	8.45		*Pan troglodytes*	588	0.17	
	92≤L≤100	*Homo sapiens*	108,257,819	64.80	7.55	*Homo sapiens*	345,879	99.52	588.23
		Pan troglodytes	14,340,123	8.58		*Pan troglodytes*	588	0.17	
	94≤L≤100	*Homo sapiens*	95,293,011	65.53	7.53	*Homo sapiens*	325,542	99.54	568.14
		Pan troglodytes	12,647,500	8.70		*Pan troglodytes*	573	0.18	

	96≤L≤100	*Homo sapiens*	73,473,822	66.88	7.82	*Homo sapiens*	280,716	99.57	559.20
		Pan troglodytes	9,398,919	8.56		*Pan troglodytes*	502	0.18	
	98≤L≤100	*Homo sapiens*	42,462,571	68.80	8.98	*Homo sapiens*	204,494	99.59	501.21
		Pan troglodytes	4,729,012	7.66		*Pan troglodytes*	408	0.20	
Villabruna	L=100	*Homo sapiens*	41,721,594	68.26	6.99	*Homo sapiens*	154,459	99.79	1514.30
		Pan troglodytes	5,967,427	9.76		*Pan troglodytes*	102	0.07	
	90≤L≤100	*Homo sapiens*	106,808,926	64.30	7.01	*Homo sapiens*	216,980	99.68	847.58
		Pan troglodytes	15,243,427	9.18		*Pan troglodytes*	256	0.12	
	92≤L≤100	*Homo sapiens*	103,800,584	64.50	6.94	*Homo sapiens*	215,003	99.69	839.86
		Pan troglodytes	14,954,416	9.29		*Pan troglodytes*	256	0.12	
	94≤L≤100	*Homo sapiens*	95,085,144	64.99	6.84	*Homo sapiens*	211,450	99.70	825.98
		Pan troglodytes	13,911,382	9.51		*Pan troglodytes*	256	0.12	
	96≤L≤100	*Homo sapiens*	76,576,760	66.23	6.80	*Homo sapiens*	203,603	99.72	795.32
		Pan troglodytes	11,267,010	9.74		*Pan troglodytes*	256	0.13	
	98≤L≤100	*Homo sapiens*	45,270,262	68.71	7.10	*Homo sapiens*	174,986	99.75	892.79
		Pan troglodytes	6,379,653	9.68		*Pan troglodytes*	196	0.11	
N1	L=100	*Mus musculus*	188,288	15.61	1.61	*Mammuthus primigenius*	222	80.14	11.68
		Homo sapiens	117,051	9.70		*Doliolum nationalis*	19	6.86	

	90≤L≤100	*Homo sapiens*	601,907	16.81	1.69	*Mammuthus primigenius*	500	57.34	7.46
		Mus musculus	355,727	9.94		*Ursus arctos*	67	7.68	
	92≤L≤100	*Homo sapiens*	435,227	14.60	1.40	*Mammuthus primigenius*	490	59.61	7.31
		Mus musculus	310,438	10.41		*Ursus arctos*	67	8.15	
	94≤L≤100	*Homo sapiens*	317,509	12.94	1.16	*Mammuthus primigenius*	431	62.10	6.43
		Mus musculus	273,166	11.14		*Ursus arctos*	67	9.65	
	96≤L≤100	*Mus musculus*	249,058	12.71	1.19	*Mammuthus primigenius*	396	65.45	5.91
		Homo sapiens	209,928	10.71		*Ursus arctos*	67	11.07	
	98≤L≤100	*Mus musculus*	203,598	14.85	1.61	*Mammuthus primigenius*	331	79.95	17.42
		Homo sapiens	126,806	9.25		*Doliolum nationalis*	19	4.59	
N2	L=100	*Mus musculus*	311,203	12.88	3.61	*Stenopirates sp.*	53	46.49	1.06
		Zebrafish DNA	86,226	3.57		*Mammuthus primigenius*	50	43.86	
	90≤L≤100	*Mus musculus*	396,387	8.76	1.33	*Mammuthus primigenius*	140	40.23	1.92
		Uncultured bacterium	298,026	6.58		*Stenopirates sp.*	73	20.98	
	92≤L≤100	*Mus musculus*	391,843	9.29	1.45	*Mammuthus primigenius*	140	47.78	1.97
		Uncultured bacterium	270,712	6.42		*Stenopirates sp.*	71	24.23	
	94≤L≤100	*Mus musculus*	381,371	9.97	1.72	*Mammuthus primigenius*	140	53.64	2.03
		Uncultured bacterium	221,651	5.80		*Stenopirates sp.*	69	26.44	

	96≤L≤100	*Mus musculus*	364,498	10.69	2.16	*Mammuthus primigenius*	136	59.39	2.03
		Uncultured bacterium	169,021	4.96		*Stenopirates sp.*	67	29.26	
	98≤L≤100	*Mus musculus*	325,866	11.83	2.99	*Mammuthus primigenius*	96	57.14	1.63
		Uncultured bacterium	109,032	3.96		*Stenopirates sp.*	59	35.12	
N3	L=100	*Mus musculus*	81,195	10.93	1.33	*Mammuthus primigenius*	292	89.30	29.20
		Homo sapiens	61,027	8.21		*Mammuthus columbi*	10	3.06	
	90≤L≤100	*Homo sapiens*	386,508	14.62	2.95	*Mammuthus primigenius*	656	67.63	12.86
		Uncultured bacterium	130,996	4.95		*Loxodonta cyclotis*	51	5.26	
	92≤L≤100	*Homo sapiens*	324,830	14.26	2.10	*Mammuthus primigenius*	656	77.63	16.82
		Uncultured bacterium	154,548	6.79		*Loxodonta cyclotis*	39	4.62	
	94≤L≤100	*Homo sapiens*	239,208	13.23	1.78	*Mammuthus primigenius*	652	81.70	20.38
		Uncultured bacterium	134,367	7.43		*Mammuthus columbi*	32	4.01	
	96≤L≤100	*Homo sapiens*	144,200	10.81	1.36	*Mammuthus primigenius*	585	86.67	22.50
		Uncultured bacterium	106,135	7.96		*Mammuthus columbi*	26	3.85	
	98≤L≤100	Uncultured bacterium	69,836	7.93	0.84	*Mammuthus primigenius*	408	89.47	25.50
		Mus musculus	83,619	9.49		*Mammuthus columbi*	16	3.51	
N6	L=100	Uncultured bacterium	76,277	20.88	4.45	*Mammuthus primigenius*	2	50.00	2.00
		Homo sapiens	17,128	4.69		*Reclinomonas americana*	1	25.00	

	90≤L≤100	Uncultured bacterium	350,999	19.41	3.12	*Reclinomonas americana*	23	18.85	1.15
		Homo sapiens	112,643	6.23		*Mammuthus primigenius*	20	16.39	
	92≤L≤100	Uncultured bacterium	320,379	20.71	3.19	*Mammuthus primigenius*	20	22.22	1.18
		Homo sapiens	100,361	6.49		*Reclinomonas americana*	17	18.89	
	94≤L≤100	Uncultured bacterium	266,273	21.83	3.21	*Mammuthus primigenius*	20	36.36	2.22
		Homo sapiens	82,884	6.79		*Reclinomonas americana*	9	16.36	
	96≤L≤100	Uncultured bacterium	193,299	22.39	3.46	*Mammuthus primigenius*	20	48.78	4.00
		Homo sapiens	55,829	6.47		*Reclinomonas americana*	5	12.20	
	98≤L≤100	Uncultured bacterium	114,502	22.38	4.11	*Mammuthus primigenius*	20	68.97	5.00
		Homo sapiens	27,856	5.45		*Reclinomonas americana*	4	13.79	
N9	L=100	Uncultured bacterium	85,802	26.68	11.73	*Mammuthus primigenius*	37	75.51	18.50
		Arthrobacter sp.	7,312	2.27		*Geococcyx californianus*	2	4.08	
	90≤L≤100	Uncultured bacterium	336,339	14.25	4.15	*Mammuthus primigenius*	180	55.90	6.21
		Bordetella pertussis	81,133	3.44		*Reclinomonas americana*	29	9.01	
	92≤L≤100	Uncultured bacterium	311,832	16.38	5.40	*Mammuthus primigenius*	180	63.16	10.59
		Streptomyces sp.	57,767	3.03		*Reclinomonas americana*	17	5.96	
	94≤L≤100	Uncultured bacterium	274,361	19.12	6.57	*Mammuthus primigenius*	180	71.43	15.00
		Streptomyces sp.	41,752	2.91		*Loxodonta cyclotis*	12	4.76	

	96≤L≤100	Uncultured bacterium	216,929	22.29	8.35	*Mammuthus primigenius*	156	75.73	13.00
		Streptomyces sp.	25,978	2.67		*Elephas maximus*	12	5.83	
	98≤L≤100	Uncultured bacterium	133,288	27.46	13.44	*Mammuthus primigenius*	127	79.87	15.88
		Arthrobacter sp.	9,916	2.04		*Elephas maximus*	8	5.03	
N12	L=100	Uncultured bacterium	137,895	8.97	1.17	*Mammuthus primigenius*	126	75.00	15.75
		Mus musculus	117,967	7.67		*Sarcophilus harrisii*	8	4.76	
	90≤L≤100	Uncultured bacterium	441,502	9.26	2.27	*Mammuthus primigenius*	400	40.94	5.26
		Pseudomonas sp.	194,697	4.08		*Sarcophilus harrisii*	76	7.78	
	92≤L≤100	Uncultured bacterium	411,562	9.94	2.28	*Mammuthus primigenius*	400	45.71	5.33
		Pseudomonas sp.	180,712	4.37		*Equus caballus*	75	8.57	
	94≤L≤100	Uncultured bacterium	372,038	10.56	2.22	*Mammuthus primigenius*	397	64.14	14.18
		Pseudomonas sp.	167,392	4.75		*Sarcophilus harrisii*	28	4.52	
	96≤L≤100	Uncultured bacterium	302,408	10.76	1.98	*Mammuthus primigenius*	351	75.97	17.55
		Pseudomonas sp.	152,753	5.44		*Sarcophilus harrisii*	20	4.33	
	98≤L≤100	Uncultured bacterium	213,025	10.69	1.63	*Mammuthus primigenius*	283	82.75	21.77
		Pseudomonas sp.	130,371	6.54		*Mammuthus columbi*	13	3.80	

‡ SR: species ranking; VMH: valid mapping hits; PoVMH: proportion of VMH; *R*: the ratio of the *PoVMH* between the top 1 species and the secondly ranked species.

Table S3. Results of BLAST search by use of animal mtDNA database with selected 3220 sequences

Samples	Similarity Ls (%)	SR	VMH	PoVMH	*R*	Similarity Ls (%)	SR	VMH	PoVMH	*R*
British aurochs	L=100	*Chrysomya albiceps*	4850	0.17	2.31					
		Geochelone pardalis	2100	0.07						
	90≤L≤100	*Chrysomya albiceps*	7117	0.06	1.03	90≤L<100	*Geochelone pardalis*	4779	0.06	1.645
		Geochelone pardalis	6879	0.06			*Andalucia godoyi*	2906	0.03	
	92≤L≤100	*Chrysomya albiceps*	6997	0.07	1.08	92≤L<100	*Geochelone pardalis*	4350	0.07	1.784
		Geochelone pardalis	6450	0.07			*Andalucia godoyi*	2439	0.04	
	94≤L≤100	*Chrysomya albiceps*	6776	0.09	1.59	94≤L<100	*Geochelone pardalis*	2172	0.05	1.128
		Geochelone pardalis	4272	0.06			*Chrysomya albiceps*	1926	0.04	
	96≤L≤100	*Chrysomya albiceps*	6120	0.11	1.8	96≤L<100	*Geochelone pardalis*	1293	0.05	1.018
		Geochelone pardalis	3393	0.06			*Chrysomya albiceps*	1270	0.05	
	98≤L≤100	*Chrysomya albiceps*	4850	0.15	2.31	98≤L<100	*Bos indicus*	189	0.06	1.027
		Geochelone pardalis	2100	0.07			*Bos taurus*	184	0.06	
JK2911	L=100	*Homo sapiens*	5E+05	0.52	22.2					
		Pan paniscus	21595	0.02						
	90≤L≤100	*Homo sapiens*	9E+05	0.16	2.58	90≤L<100	*Homo sapiens*	5E+05	0.09	1.333
		Pan paniscus	4E+05	0.06			*Pan paniscus*	3E+05	0.07	
	92≤L≤100	*Homo sapiens*	9E+05	0.20	3.7	92≤L<100	*Homo sapiens*	4E+05	0.12	1.937
		Pan troglodytes	3E+05	0.06			*Pan troglodytes*	2E+05	0.06	

	94≤L≤100	*Homo sapiens*	9E+05	0.26	5.89	94≤L<100	*Homo sapiens*	4E+05	0.17	3.196
		Pan troglodytes	2E+05	0.04			*Pan troglodytes*	1E+05	0.05	
	96≤L≤100	*Homo sapiens*	9E+05	0.36	10.3	96≤L<100	*Homo sapiens*	4E+05	0.25	6.077
		Pan paniscus	83121	0.03			*Pan paniscus*	61526	0.04	
	98≤L≤100	*Homo sapiens*	7E+05	0.52	19.2	98≤L<100	*Homo sapiens*	2E+05	0.51	14.81
		Pan paniscus	36442	0.03			*Pan paniscus*	14847	0.03	
Direkli5	L=100	*Capra caucasica*	4000	0.07	1.57					
		Capra hircus	2552	0.04						
	90≤L≤100	*Capra caucasica*	9157	0.02	1.08	90≤L<100	*Capra ibex*	6158	0.02	1.005
		Capra falconeri	8509	0.02			*Capra nubiana*	6127	0.02	
	92≤L≤100	*Capra caucasica*	8882	0.03	1.1	92≤L<100	*Capra falconeri*	5678	0.02	1.005
		Capra falconeri	8084	0.03			*Capra ibex*	5648	0.02	
	94≤L≤100	*Capra caucasica*	8394	0.04	1.14	94≤L<100	*Capra falconeri*	4986	0.03	1.05
		Capra falconeri	7392	0.03			*Capra ibex*	4748	0.03	
	96≤L≤100	*Capra caucasica*	7612	0.05	1.25	96≤L<100	*Capra falconeri*	3696	0.04	1.023
		Capra falconeri	6102	0.04			*Capra caucasica*	3612	0.04	
	98≤L≤100	*Capra caucasica*	5582	0.07	1.47	98≤L<100	*Capra caucasica*	1582	0.08	1.229
		Capra hircus	3800	0.05			*Capra falconeri*	1287	0.07	
Ancient horse	L=100	*Andalucia godoyi*	230	0.21	1.01					
		Chrysomya albiceps	228	0.20						

	90≤L≤100	*Andalucia godoyi*	1399	0.14	1.02	90≤L<100	*Jakoba bahamiensis*	1329	0.15	1.137
		Jakoba bahamiensis	1375	0.14			*Andalucia godoyi*	1169	0.13	
	92≤L≤100	*Andalucia godoyi*	1154	0.15	1.09	92≤L<100	*Jakoba bahamiensis*	1017	0.16	1.101
		Jakoba bahamiensis	1063	0.14			*Andalucia godoyi*	924	0.14	
	94≤L≤100	*Andalucia godoyi*	839	0.17	1.32	94≤L<100	*Andalucia godoyi*	609	0.16	1.034
		Jakoba bahamiensis	635	0.13			*Jakoba bahamiensis*	589	0.15	
	96≤L≤100	*Andalucia godoyi*	521	0.19	1.54	96≤L<100	*Andalucia godoyi*	291	0.17	1.017
		Chrysomya albiceps	339	0.12			*Jakoba bahamiensis*	286	0.17	
	98≤L≤100	*Andalucia godoyi*	291	0.21	1.28	98≤L<100	*Andalucia godoyi*	61	0.25	1.452
		Chrysomya albiceps	228	0.17			*Jakoba bahamiensis*	42	0.17	
AfontovaGora3	L=100	*Homo sapiens*	6392	0.51	11.4					
		Geochelone pardalis	563	0.04						
	90≤L≤100	*Homo sapiens*	16297	0.16	2.85	90≤L<100	*Homo sapiens*	9905	0.11	1.814
		Pan troglodytes	5712	0.06			*Pan troglodytes*	5461	0.06	
	92≤L≤100	*Homo sapiens*	16056	0.21	4.01	92≤L<100	*Homo sapiens*	9664	0.15	2.577
		Pan troglodytes	4001	0.05			*Pan troglodytes*	3750	0.06	
	94≤L≤100	*Homo sapiens*	15446	0.27	6.3	94≤L<100	*Homo sapiens*	9054	0.21	4.114
		Pan troglodytes	2452	0.04			*Pan troglodytes*	2201	0.05	
	96≤L≤100	*Homo sapiens*	13937	0.37	11.3	96≤L<100	*Homo sapiens*	7545	0.30	7.802
		Pan paniscus	1237	0.03			*Pan paniscus*	967	0.04	
	98≤L≤100	*Homo sapiens*	10018	0.52	17.8	98≤L<100	*Homo sapiens*	3626	0.54	15.24

		Geochelone pardalis	563	0.03			*Pan paniscus*	238	0.04	
Villabruna	L=100	*Chrysomya albiceps*	7630	0.18	1.08					
		Geochelone pardalis	7069	0.17						
	90≤L≤100	*Chrysomya albiceps*	13539	0.11	1.06	90≤L<100	*Chrysomya albiceps*	5909	0.07	1.036
		Geochelone pardalis	12771	0.10			*Geochelone pardalis*	5702	0.07	
	92≤L≤100	*Chrysomya albiceps*	13042	0.12	1.07	92≤L<100	*Chrysomya albiceps*	5412	0.08	1.057
		Geochelone pardalis	12191	0.11			*Geochelone pardalis*	5122	0.08	
	94≤L≤100	*Chrysomya albiceps*	12303	0.13	1.08	94≤L<100	*Chrysomya albiceps*	4673	0.10	1.085
		Geochelone pardalis	11374	0.12			*Geochelone pardalis*	4305	0.09	
	96≤L≤100	*Chrysomya albiceps*	10431	0.15	1.08	96≤L<100	*Chrysomya albiceps*	2801	0.10	1.051
		Geochelone pardalis	9670	0.14			*Homo sapiens*	2664	0.09	
	98≤L≤100	*Homo sapiens*	7793	0.16	1.02	98≤L<100	*Homo sapiens*	1142	0.23	7.664
		Chrysomya albiceps	7635	0.16			*Pan paniscus*	149	0.03	
N1	L=100	*Campodea lubbocki*	2099	0.29	1.63					
		Doliolum nationalis	1290	0.18						
	90≤L≤100	*Campodea lubbocki*	4176	0.14	1.53	90≤L<100	*Campodea lubbocki*	2077	0.09	1.27
		Doliolum nationalis	2723	0.09			*Elephas maximus*	1636	0.07	
	92≤L≤100	*Campodea lubbocki*	3756	0.15	1.54	92≤L<100	*Campodea lubbocki*	1657	0.10	1.224

	94≤L≤100	*Doliolum nationalis*	2435	0.10	1.6	94≤L<100	*Mammuthus columbi*	1354	0.08	1.117
		Campodea lubbocki	3487	0.17			*Campodea lubbocki*	1388	0.11	
		Doliolum nationalis	2181	0.11			*Mammuthus columbi*	1243	0.10	
	96≤L≤100	*Campodea lubbocki*	2792	0.20	1.67	96≤L<100	*Mammuthus columbi*	1066	0.16	1.025
		Mammuthus primigenius	1668	0.12			*Mammuthus primigenius*	1040	0.16	
	98≤L≤100	*Campodea lubbocki*	2099	0.23	1.63	98≤L<100	*Mammuthus columbi*	668	0.37	1.014
		Doliolum nationalis	1290	0.14			*Mammuthus primigenius*	659	0.37	
N2	L=100	*Stenopirates sp.*	4137	0.79	15.9					
		Mammuthus primigenius	260	0.05						
	90≤L≤100	*Stenopirates sp.*	5966	0.32	5.98	90≤L<100	*Stenopirates sp.*	1829	0.14	1.872
		Andalucia godoyi	998	0.05			*Andalucia godoyi*	977	0.07	
	92≤L≤100	*Stenopirates sp.*	5863	0.40	6.51	92≤L<100	*Stenopirates sp.*	1726	0.18	2.659
		Mammuthus primigenius	901	0.06			*Mammuthus columbi*	649	0.07	
	94≤L≤100	*Stenopirates sp.*	5649	0.48	6.55	94≤L<100	*Stenopirates sp.*	1512	0.23	2.507
		Mammuthus primigenius	862	0.07			*Mammuthus columbi*	603	0.09	
	96≤L≤100	*Stenopirates sp.*	5242	0.59	6.73	96≤L<100	*Stenopirates sp.*	1105	0.30	2.129
		Mammuthus primigenius	779	0.09			*Mammuthus primigenius*	519	0.14	
	98≤L≤100	*Stenopirates sp.*	4464	0.70	8.25	98≤L<100	*Stenopirates sp.*	327	0.28	1.164

		Mammuthus primigenius	541	0.08			*Mammuthus primigenius*	281	0.24	
N3	L=100	*Mammuthus primigenius*	1589	0.24	1.02					
		Mammuthus columbi	1564	0.24						
	90≤L≤100	*Mammuthus primigenius*	4296	0.11	1	90≤L<100	*Elephas maximus*	3206	0.09	1.017
		Mammuthus columbi	4286	0.11			*Loxodonta cyclotis*	3153	0.09	
	92≤L≤100	*Mammuthus primigenius*	4189	0.14	1	92≤L<100	*Elephas maximus*	2736	0.12	1.02
		Mammuthus columbi	4185	0.14			*Loxodonta cyclotis*	2683	0.12	
	94≤L≤100	*Mammuthus primigenius*	4033	0.18	1.01	94≤L<100	*Mammuthus primigenius*	2444	0.15	1.002
		Mammuthus columbi	4004	0.18			*Mammuthus columbi*	2440	0.15	
	96≤L≤100	*Mammuthus primigenius*	3654	0.22	1.02	96≤L<100	*Mammuthus primigenius*	2065	0.20	1.017
		Mammuthus columbi	3595	0.22			*Mammuthus columbi*	2031	0.20	
	98≤L≤100	*Mammuthus primigenius*	2783	0.27	1.03	98≤L<100	*Mammuthus primigenius*	1194	0.30	1.059
		Mammuthus columbi	2692	0.26			*Mammuthus columbi*	1128	0.29	
N6	L=100	*Seculamonas ecuadoriensis*	52	0.10	1.24					
		Adoxophyes honmai	42	0.08						
	90≤L≤100	*Andalucia godoyi*	969	0.14	1.13	90≤L<100	*Andalucia godoyi*		0.15	1.137
		Reclinomonas americana	854	0.12			*Reclinomonas americana*	833	0.13	
	92≤L≤100	*Andalucia godoyi*	642	0.14	1.16	92≤L<100	*Andalucia godoyi*	620	0.15	1.165

		Reclinomonas americana	553	0.12			*Reclinomonas americana*	532	0.13	
	94≤L≤100	*Andalucia godoyi*	379	0.13	1.16	94≤L<100	*Andalucia godoyi*	357	0.15	1.17
		Reclinomonas americana	326	0.11			*Reclinomonas americana*	305	0.13	
	96≤L≤100	*Reclinomonas americana*	150	0.10	1	96≤L<100	*Reclinomonas americana*	129	0.13	1.008
		Andalucia godoyi	150	0.10			*Andalucia godoyi*	128	0.12	
	98≤L≤100	*Mammuthus primigenius*	87	0.12	1.12	98≤L<100	*Mammuthus primigenius*	57	0.24	1.267
		Mammuthus columbi	78	0.10			*Mammuthus columbi*	45	0.19	
N9	L=100	*Mammuthus primigenius*	165	0.17	1.07					
		Mammuthus columbi	154	0.16						
	90≤L≤100	*Andalucia godoyi*	1129	0.10	1.08	90≤L<100	*Andalucia godoyi*	1104	0.11	1.079
		Reclinomonas americana	1042	0.09			*Reclinomonas americana*	1023	0.10	
	92≤L≤100	*Andalucia godoyi*	744	0.09	1.03	92≤L<100	*Andalucia godoyi*	719	0.10	1.035
		Seculamonas ecuadoriensis	720	0.09			*Seculamonas ecuadoriensis*	695	0.10	
	94≤L≤100	*Mammuthus primigenius*	526	0.11	1	94≤L<100	*Andalucia godoyi*	427	0.11	1.154
		Mammuthus columbi	524	0.11			*Mammuthus columbi*	370	0.09	
	96≤L≤100	*Mammuthus primigenius*	473	0.16	1.02	96≤L<100	*Mammuthus columbi*	310	0.16	1.006
		Mammuthus columbi	464	0.16			*Mammuthus primigenius*	308	0.16	
	98≤L≤100	*Mammuthus primigenius*	334	0.21	1.02	98≤L<100	*Mammuthus columbi*	173	0.28	1.024

		Mammuthus columbi	327	0.20			*Mammuthus primigenius*	169	0.27	
N12	L=100	*Campodea lubbocki*	1028	0.21	1.62					
		Mammuthus primigenius	634	0.13						
	90≤L≤100	*Mammuthus primigenius*	2165	0.07	1	90≤L<100	*Elephas maximus*	1739	0.06	1.039
		Mammuthus columbi	2165	0.07			*Loxodonta cyclotis*	1673	0.06	
	92≤L≤100	*Mammuthus primigenius*	2127	0.09	1	92≤L<100	*Mammuthus columbi*	1500	0.08	1.005
		Mammuthus columbi	2121	0.09			*Mammuthus primigenius*	1493	0.08	
	94≤L≤100	*Mammuthus primigenius*	2042	0.11	1	94≤L<100	*Mammuthus columbi*	1412	0.11	1.003
		Mammuthus columbi	2033	0.11			*Mammuthus primigenius*	1408	0.11	
	96≤L≤100	*Mammuthus primigenius*	1844	0.15	1.01	96≤L<100	*Mammuthus primigenius*	1210	0.16	1.002
		Mammuthus columbi	1829	0.15			*Mammuthus columbi*	1208	0.16	
	98≤L≤100	*Mammuthus primigenius*	1380	0.18	1.03	98≤L<100	*Mammuthus primigenius*	746	0.27	1.039
		Mammuthus columbi	1339	0.17			*Mammuthus columbi*	718	0.26	

‡ SR: species ranking; VMH: valid mapping hits; PoVMH: proportion of VMH; R: the ratio of the *PoVMH* between the top 1 species and the secondly ranked species.

Table S4. Results of BLAST search by use of whole animal mtDNA database

**Samples**	**Similarity Ls (%)**	**SR**	**VMH**	**PoVMH (%)**	***R***	**Similarity Ls (%)**	**SR**	**VMH**	**PoVMH (%)**	***R***
British aurochs	L=100	*Bos taurus*	329165	0.82	20.24					
		Bos grunniens	16265	0.04						
	90≤L≤100	*Bos taurus*	443353	0.74	17.85	90≤L<100	*Bos taurus*	114188	0.59	7.09
		Bos grunniens	24843	0.04			*Sarcophilus harrisii*	16111	0.08	
	92≤L≤100	*Bos taurus*	440680	0.75	18.32	92≤L<100	*Bos taurus*	111515	0.61	7.17
		Bos grunniens	24060	0.04			*Sarcophilus harrisii*	15557	0.09	
	94≤L≤100	*Bos taurus*	432895	0.77	18.84	94≤L<100	*Bos taurus*	103730	0.65	7.19
		Bos grunniens	22972	0.04			*Sarcophilus harrisii*	14424	0.09	
	96≤L≤100	*Bos taurus*	420270	0.79	19.78	96≤L<100	*Bos taurus*	91105	0.71	10.28
		Bos indicus	21251	0.04			*Sarcophilus harrisii*	8865	0.07	
	98≤L≤100	*Bos taurus*	369572	0.82	20.71	98≤L<100	*Bos taurus*	40407	0.85	21.01
		Bos grunniens	17844	0.04			*Bos indicus*	1923	0.04	
JK2911	L=100	*Homo sapiens*	35462589	1.00	1781.95					
		Pan troglodytes	19901	0.00						
	90≤L≤100	*Homo sapiens*	56323464	1.00	1537.21	90≤L<100	*Homo sapiens*	20860875	1.00	1246.24
		Pan troglodytes	36640	0.00			*Pan troglodytes*	16739	0.00	
	92≤L≤100	*Homo sapiens*	56209448	1.00	1554.42	92≤L<100	*Homo sapiens*	20746859	1.00	1275.94
		Pan troglodytes	36161	0.00			*Pan troglodytes*	16260	0.00	
	94≤L≤100	*Homo sapiens*	55939854	1.00	1625.07	94≤L<100	*Homo sapiens*	20477265	1.00	1410.09
		Pan troglodytes	34423	0.00			*Pan troglodytes*	14522	0.00	
	96≤L≤100	*Homo sapiens*	54614260	1.00	1733.13	96≤L<100	*Homo sapiens*	19151671	1.00	1649.44
		Pan troglodytes	31512	0.00			*Pan troglodytes*	11611	0.00	
	98≤L≤100	*Homo sapiens*	48532486	1.00	1965.91	98≤L<100	*Homo sapiens*	13069897	1.00	2730.86

		Pan troglodytes	24687	0.00			*Pan troglodytes*	4786	0.00	
Direkli5	L=100	*Capra hircus*	27033	0.19	1.44					
		Bos taurus	18712	0.13						
	90≤L≤100	*Bos taurus*	132649	0.17	1.31	90≤L<100	*Bos taurus*	113937	0.18	1.54
		Capra hircus	100971	0.13			*Capra hircus*	73938	0.12	
	92≤L≤100	*Bos taurus*	100449	0.16	1.07	92≤L<100	*Bos taurus*	81737	0.16	1.22
		Capra hircus	94034	0.15			*Capra hircus*	67001	0.14	
	94≤L≤100	*Capra hircus*	84025	0.17	1.23	94≤L<100	*Capra hircus*	56992	0.17	1.15
		Bos taurus	68064	0.14			*Bos taurus*	49352	0.15	
	96≤L≤100	*Capra hircus*	70124	0.21	1.57	96≤L<100	*Capra hircus*	43091	0.22	1.66
		Bos taurus	44707	0.13			*Bos taurus*	25995	0.13	
	98≤L≤100	*Capra hircus*	43241	0.22	1.81	98≤L<100	*Capra hircus*	16208	0.29	3.10
		Bos taurus	23942	0.12			*Bos taurus*	5230	0.09	
Ancient horse	L=100	*Equus caballus*	10393	0.80	10.39					
		Homo sapiens	1000	0.08						
	90≤L≤100	*Equus caballus*	19718	0.51	5.44	90≤L<100	*Equus caballus*	9325	0.36	2.67
		Reclinomonas americana	3624	0.09			*Reclinomonas americana*	3486	0.14	
	92≤L≤100	*Equus caballus*	19632	0.57	7.38	92≤L<100	*Equus caballus*	9239	0.43	3.66
		Reclinomonas americana	2659	0.08			*Reclinomonas americana*	2521	0.12	
	94≤L≤100	*Equus caballus*	18967	0.66	12.69	94≤L<100	*Equus caballus*	8574	0.55	6.32
		Reclinomonas americana	1495	0.05			*Reclinomonas americana*	1357	0.09	
	96≤L≤100	*Equus caballus*	17219	0.74	17.22	96≤L<100	*Equus caballus*	6826	0.67	11.55
		Homo sapiens	1000	0.04			*Reclinomonas americana*	591	0.06	
	98≤L≤100	*Equus caballus*	14714	0.82	14.71	98≤L<100	*Equus caballus*	4321	0.89	37.25
		Homo sapiens	1000	0.06			*Equus przewalskii*	116	0.02	
AfontovaGora3	L=100	*Homo sapiens*	1420059	1.00	647.84					
		Pan troglodytes	2192	0.00						
	90≤L≤100	*Homo sapiens*	4057259	1.00	780.24	90≤L<100	*Homo sapiens*	2637200	1.00	609.90

		Sarcophilus harrisii	5200	0.00			*Sarcophilus harrisii*	4324	0.00	
	92≤L≤100	*Homo sapiens*	3988477	1.00	918.16	92≤L<100	*Homo sapiens*	2568418	1.00	740.60
		Sarcophilus harrisii	4344	0.00			*Sarcophilus harrisii*	3468	0.00	
	94≤L≤100	*Homo sapiens*	3804427	1.00	997.49	94≤L<100	*Homo sapiens*	2384368	1.00	1006.06
		Pan troglodytes	3814	0.00			*Sarcophilus harrisii*	2370	0.00	
	96≤L≤100	*Homo sapiens*	3360442	1.00	967.31	96≤L<100	*Homo sapiens*	1940383	1.00	1513.56
		Pan troglodytes	3474	0.00			*Pan troglodytes*	1282	0.00	
	98≤L≤100	*Homo sapiens*	2398134	1.00	946.38	98≤L<100	*Homo sapiens*	978075	1.00	2859.87
		Pan troglodytes	2534	0.00			*Pan troglodytes*	342	0.00	
Villabruna	L=100	*Homo sapiens*	1641905	0.98	151.20					
		Sarcophilus harrisii	10859	0.01						
	90≤L≤100	*Homo sapiens*	2390295	0.97	65.47	90≤L<100	*Homo sapiens*	748390	0.94	29.18
		Sarcophilus harrisii	36508	0.01			*Sarcophilus harrisii*	25649	0.03	
	92≤L≤100	*Homo sapiens*	2367275	0.97	70.61	92≤L<100	*Homo sapiens*	725370	0.95	32.00
		Sarcophilus harrisii	33524	0.01			*Sarcophilus harrisii*	22665	0.03	
	94≤L≤100	*Homo sapiens*	2330725	0.97	84.66	94≤L<100	*Homo sapiens*	688820	0.96	41.32
		Sarcophilus harrisii	27529	0.01			*Sarcophilus harrisii*	16670	0.02	
	96≤L≤100	*Homo sapiens*	2240322	0.98	111.26	96≤L<100	*Homo sapiens*	598417	0.97	64.51
		Sarcophilus harrisii	20136	0.01			*Sarcophilus harrisii*	9277	0.02	
	98≤L≤100	*Homo sapiens*	1910294	0.98	175.26	98≤L<100	*Homo sapiens*	268389	1.00	1024.39
		Sarcophilus harrisii	10900	0.01			*Pan troglodytes*	262	0.00	
N1	L=100	*Mammuthus primigenius*	15963	0.70	9.76					
		Campodea lubbocki	1635	0.07						

	90≤L≤100	*Mammuthus primigenius*	41877	0.51	7.80	90≤L<100	*Mammuthus primigenius*	25914	0.43	5.22
		Sarcophilus harrisii	5366	0.06			*Sarcophilus harrisii*	4966	0.08	
	92≤L≤100	*Mammuthus primigenius*	40508	0.55	9.87	92≤L<100	*Mammuthus primigenius*	24545	0.49	6.62
		Sarcophilus harrisii	4106	0.06			*Sarcophilus harrisii*	3706	0.07	
	94≤L≤100	*Mammuthus primigenius*	38348	0.60	16.64	94≤L<100	*Mammuthus primigenius*	22385	0.55	11.28
		Sarcophilus harrisii	2304	0.04			*Elephas maximus*	1985	0.05	
	96≤L≤100	*Mammuthus primigenius*	34593	0.67	17.61	96≤L<100	*Mammuthus primigenius*	18630	0.64	16.75
		Campodea lubbocki	1964	0.04			*Elephas maximus*	1112	0.04	
	98≤L≤100	*Mammuthus primigenius*	27488	0.73	16.81	98≤L<100	*Mammuthus primigenius*	11525	0.78	16.61
		Campodea lubbocki	1635	0.04			*Mammuthus columbi*	694	0.05	
N2	L=100	*Mammuthus primigenius*	6624	0.56	1.67					
		Stenopirates sp.	3960	0.34						
	90≤L≤100	*Mammuthus primigenius*	18975	0.44	3.37	90≤L<100	*Mammuthus primigenius*	12351	0.40	4.86
		Stenopirates sp.	5627	0.13			*Reclinomonas americana*	2542	0.08	
	92≤L≤100	*Mammuthus primigenius*	18676	0.50	3.37	92≤L<100	*Mammuthus primigenius*	12052	0.48	7.59
		Stenopirates sp.	5547	0.15			*Stenopirates sp.*	1587	0.06	
	94≤L≤100	*Mammuthus primigenius*	17860	0.57	3.33	94≤L<100	*Mammuthus primigenius*	11236	0.57	8.04
		Stenopirates sp.	5358	0.17			*Stenopirates sp.*	1398	0.07	
	96≤L≤100	*Mammuthus primigenius*	15976	0.62	3.20	96≤L<100	*Mammuthus primigenius*	9352	0.67	9.06

		Stenopirates sp.	4992	0.19			*Stenopirates sp.*	1032	0.07	
	98≤L≤100	*Mammuthus primigenius*	11811	0.65	2.76	98≤L<100	*Mammuthus primigenius*	5187	0.81	15.96
		Stenopirates sp.	4285	0.24			*Stenopirates sp.*	325	0.05	
N3	L=100	*Mammuthus primigenius*	37196	0.82	15.13					
		Homo sapiens	2459	0.05						
	90≤L≤100	*Mammuthus primigenius*	86297	0.57	12.18	90≤L<100	*Mammuthus primigenius*	49101	0.47	7.63
		Elephas maximus	7085	0.05			*Elephas maximus*	6436	0.06	
	92≤L≤100	*Mammuthus primigenius*	84883	0.64	13.74	92≤L<100	*Mammuthus primigenius*	47687	0.55	8.62
		Elephas maximus	6178	0.05			*Elephas maximus*	5529	0.06	
	94≤L≤100	*Mammuthus primigenius*	81917	0.71	17.66	94≤L<100	*Mammuthus primigenius*	44721	0.64	11.21
		Elephas maximus	4639	0.04			*Elephas maximus*	3990	0.06	
	96≤L≤100	*Mammuthus primigenius*	75114	0.78	20.49	96≤L<100	*Mammuthus primigenius*	37918	0.74	16.88
		Mammuthus columbi	3665	0.04			*Elephas maximus*	2246	0.04	
	98≤L≤100	*Mammuthus primigenius*	58657	0.82	20.19	98≤L<100	*Mammuthus primigenius*	21461	0.83	18.42
		Homo sapiens	2905	0.04			*Mammuthus columbi*	1165	0.04	
N6	L=100	*Mammuthus primigenius*	790	0.60	16.12					
		Seculamonas ecuadoriensis	49	0.04						
	90≤L≤100	*Mammuthus primigenius*	3539	0.25	1.22	90≤L<100	*Reclinomonas americana*	2865	0.23	1.04
		Reclinomonas americana	2909	0.21			*Mammuthus primigenius*	2749	0.22	

	92≤L≤100	*Mammuthus primigenius*	3453	0.32	1.85	92≤L<100	*Mammuthus primigenius*	2663	0.28	1.46
		Reclinomonas americana	1868	0.17			*Reclinomonas americana*	1824	0.19	
	94≤L≤100	*Mammuthus primigenius*	3355	0.43	4.46	94≤L<100	*Mammuthus primigenius*	2565	0.40	3.62
		Reclinomonas americana	752	0.10			*Reclinomonas americana*	708	0.11	
	96≤L≤100	*Mammuthus primigenius*	2760	0.53	8.39	96≤L<100	*Mammuthus primigenius*	1970	0.51	6.91
		Reclinomonas americana	329	0.06			*Reclinomonas americana*	285	0.07	
	98≤L≤100	*Mammuthus primigenius*	1656	0.64	17.07	98≤L<100	*Mammuthus primigenius*	866	0.68	11.10
		Reclinomonas americana	97	0.04			*Bos grunniens*	78	0.06	
N9	L=100	*Mammuthus primigenius*	3887	0.75	15.36					
		Homo sapiens	253	0.05						
	90≤L≤100	*Mammuthus primigenius*	12048	0.41	3.52	90≤L<100	*Mammuthus primigenius*	8161	0.34	2.43
		Reclinomonas americana	3427	0.12			*Reclinomonas americana*	3360	0.14	
	92≤L≤100	*Mammuthus primigenius*	11775	0.51	5.79	92≤L<100	*Mammuthus primigenius*	7888	0.44	4.01
		Reclinomonas americana	2034	0.09			*Reclinomonas americana*	1967	0.11	
	94≤L≤100	*Mammuthus primigenius*	11091	0.62	12.43	94≤L<100	*Mammuthus primigenius*	7204	0.56	8.73
		Reclinomonas americana	892	0.05			*Reclinomonas americana*	825	0.06	
	96≤L≤100	*Mammuthus primigenius*	9953	0.71	20.31	96≤L<100	*Mammuthus primigenius*	6066	0.69	16.90
		Mammuthus columbi	490	0.04			*Elephas maximus*	359	0.04	
	98≤L≤100	*Mammuthus primigenius*	7205	0.78	20.70	98≤L<100	*Mammuthus primigenius*	3318	0.82	18.43

		Mammuthus columbi	348	0.04			*Mammuthus columbi*	180	0.04	
N12	L=100	*Mammuthus primigenius*	17030	0.79	22.17					
		Campodea lubbocki	768	0.04						
	90≤L≤100	*Mammuthus primigenius*	44271	0.49	9.27	90≤L<100	*Mammuthus primigenius*	27241	0.40	6.08
		Sarcophilus harrisii	4775	0.05			*Reclinomonas americana*	4480	0.07	
	92≤L≤100	*Mammuthus primigenius*	43562	0.57	11.43	92≤L<100	*Mammuthus primigenius*	26532	0.48	7.63
		Sarcophilus harrisii	3811	0.05			*Sarcophilus harrisii*	3477	0.06	
	94≤L≤100	*Mammuthus primigenius*	42091	0.65	17.83	94≤L<100	*Mammuthus primigenius*	25061	0.58	11.69
		Elephas maximus	2361	0.04			*Elephas maximus*	2144	0.05	
	96≤L≤100	*Mammuthus primigenius*	38192	0.73	20.39	96≤L<100	*Mammuthus primigenius*	21162	0.69	17.30
		Mammuthus columbi	1873	0.04			*Mammuthus columbi*	1223	0.04	
	98≤L≤100	*Mammuthus primigenius*	29995	0.80	21.59	98≤L<100	*Mammuthus primigenius*	12965	0.82	17.54
		Mammuthus columbi	1389	0.04			*Mammuthus columbi*	739	0.05	

‡ SR: species ranking; VMH: valid mapping hits; PoVMH: proportion of VMH; R: the ratio of the *PoVMH* between the top 1 species and the secondly ranked species.

Table S5. Analysis of One-Way ANOVA with LSD for *R* values of different similarities by use of whole animal mtDNA database based on BLAST search

Similarity Ls		Mean difference (I-J)	Std.Error	Sig.	95% Confidence Interval	
					Lower Bound	Upper Bound
90≤L≤100	92≤L≤100	-1.20517	3.24483	.712	-7.7293	5.3190
	94≤L≤100	-4.79596	3.24483	.146	-11.3201	1.7282
	96≤L≤100	-7.44488*	3.24483	.026	-13.9691	-.9207
	98≤L≤100	-8.26684*	3.24483	.014	-14.7910	-1.7427
	L=100	-5.59304	3.24483	.091	-12.1172	.9311
92≤L≤100	90≤L≤100	1.20517	3.24483	.712	-5.3190	7.7293
	94≤L≤100	-3.59079	3.24483	.274	-10.1150	2.9334
	96≤L≤100	-6.23972	3.24483	.060	-12.7639	.2845
	98≤L≤100	-7.06167*	3.24483	.034	-13.5858	-.5375
	L=100	-4.38788	3.24483	.183	-10.9120	2.1363
94≤L≤100	90≤L≤100	4.79596	3.24483	.146	-1.7282	11.3201
	92≤L≤100	3.59079	3.24483	.274	-2.9334	10.1150
	96≤L≤100	-2.64893	3.24483	.418	-9.1731	3.8752
	98≤L≤100	-3.47088	3.24483	.290	-9.9951	3.0533
	L=100	-.79709	3.24483	.807	-7.3213	5.7271
96≤L≤100	90≤L≤100	7.44488*	3.24483	.026	.9207	13.9691
	92≤L≤100	6.23972	3.24483	.060	-.2845	12.7639
	94≤L≤100	2.64893	3.24483	.418	-3.8752	9.1731
	98≤L≤100	-.82196	3.24483	.801	-7.3461	5.7022
	L=100	1.85184	3.24483	.571	-4.6723	8.3760
98≤L≤100	90≤L≤100	8.26684*	3.24483	.014	1.7427	14.7910
	92≤L≤100	7.06167*	3.24483	.034	.5375	13.5858
	94≤L≤100	3.47088	3.24483	.290	-3.0533	9.9951
	96≤L≤100	.82196	3.24483	.801	-5.7022	7.3461
	L=100	2.67380	3.24483	.414	-3.8504	9.1980
L=100	90≤L≤100	5.59304	3.24483	.091	-.9311	12.1172
	92≤L≤100	4.38788	3.24483	.183	-2.1363	10.9120
	94≤L≤100	.79709	3.24483	.807	-5.7271	7.3213
	96≤L≤100	-1.85184	3.24483	.571	-8.3760	4.6723
	98≤L≤100	-2.67380	3.24483	.414	-9.1980	3.8504

‡ The mean difference is significant at the 0.05 level.

Table S6. Analysis of One-Way ANOVA with LSD for R values of different similarities (without 100%) by use of whole animal mtDNA database based on BLAST search

Similarity Ls (%)		Mean difference (I-J)	Std.Error	Sig.	95% Confidence Interval	
					Lower Bound	Upper Bound
90≤L<100	92≤L<100	-1.04828	2.48597	.676	-6.0726	3.9761
	94≤L<100	-3.40775	2.48597	.178	-8.4321	1.6166
	96≤L<100	-7.63709*	2.48597	.004	-12.6614	-2.6128
	98≤L<100	-13.42979*	2.48597	.000	-18.4541	-8.4055
92≤L<100	90≤L<100	1.04828	2.48597	.676	-3.9761	6.0726
	94≤L<100	-2.35946	2.48597	.348	-7.3838	2.6649
	96≤L<100	-6.58881*	2.48597	.011	-11.6131	-1.5645
	98≤L<100	-12.38151*	2.48597	.000	-17.4058	-7.3572
94≤L<100	90≤L<100	3.40775	2.48597	.178	-1.6166	8.4321
	92≤L<100	2.35946	2.48597	.348	-2.6649	7.3838
	96≤L<100	-4.22934	2.48597	.097	-9.2537	.7950
	98≤L<100	-10.02204*	2.48597	.000	-15.0464	-4.9977
96≤L<100	90≤L<100	7.63709*	2.48597	.004	2.6128	12.6614
	92≤L<100	6.58881*	2.48597	.011	1.5645	11.6131
	94≤L<100	4.22934	2.48597	.097	-.7950	9.2537
	98≤L<100	-5.79270*	2.48597	.025	-10.8170	-.7684
98≤L<100	90≤L<100	13.42979*	2.48597	.000	8.4055	18.4541
	92≤L<100	12.38151*	2.48597	.000	7.3572	17.4058
	94≤L<100	10.02204*	2.48597	.000	4.9977	15.0464
	96≤L<100	5.79270*	2.48597	.025	.7684	10.8170

‡ The mean difference is significant at the 0.05 level.

Table S7. Comparisons of species identification under different query coverages by use of BLAST search based on whole animal mtDNA database

**Species**	**Coverage (*C*, %)**	**The top1 species**	**VMH**	**PoVMH of top1 species**	**PoVMH of secondly ranked species**	***R***
British aurochs	C < 90	*Bos taurus*	137	0.30	0.16	1.83
	90 ≤ C < 92	*Bos taurus*	146	0.54	0.27	2.00
	92 ≤ C < 94	*Bos taurus*	680	0.85	0.03	26.15
	94 ≤ C < 96	*Bos taurus*	473	0.79	0.04	17.52
	96 ≤ C < 98	*Bos taurus*	605	0.69	0.09	7.56
	98 ≤ C < 100	*Bos taurus*	317	0.43	0.32	1.34
	98 ≤ C ≤ 100	*Bos taurus*	33476	0.86	0.04	20.95
	C = 100	*Bos taurus*	33159	0.87	0.04	20.95
Direkli5	C < 90	*Capra hircus*	584	0.32	0.11	2.91
	90 ≤ C < 92	*Bos taurus*	45	0.27	0.25	1.07
	92 ≤ C < 94	*Capra hircus*	79	0.71	0.07	9.88
	94 ≤ C < 96	*Capra hircus*	179	0.42	0.25	1.67
	96 ≤ C < 98	*Capra hircus*	56	0.39	0.13	2.95
	98 ≤ C < 100	*Capra hircus*	193	0.19	0.19	1.02
	98 ≤ C ≤ 100	*Capra hircus*	13378	0.28	0.10	2.71
	C = 100	*Capra hircus*	13185	0.28	0.10	2.78
Ancient Horse	C < 90	*Andalucia godoyi*	9	0.26	0.18	1.50
	90 ≤ C < 92	*Equus caballus*	204	0.95	0.03	29.14
	92 ≤ C < 94	*Equus caballus*	131	0.88	0.05	16.38
	94 ≤ C < 96	*Reclinomonas americana*	6	0.43	0.21	2.00
	96 ≤ C < 98	*Reclinomonas americana*	4	0.57	0.29	2.00
	98 ≤ C < 100	*Equus caballus*	272	0.91	0.02	38.86
	98 ≤ C ≤ 100	*Equus caballus*	3950	0.91	0.02	37.98
	C = 100	*Equus caballus*	3678	0.90	0.02	37.92
N1	C < 90	*Theileria cervi*	693	0.49	0.11	4.44
	90 ≤ C < 92	*Mammuthus primigenius*	17	0.94	0.06	17.00
	92 ≤ C < 94	*Mammuthus primigenius*	49	0.89	0.07	12.25
	94 ≤ C < 96	*Mammuthus primigenius*	161	0.88	0.04	20.13
	96 ≤ C < 98	*Mammuthus primigenius*	275	0.47	0.43	1.10
	98 ≤ C < 100	*Mammuthus primigenius*	319	0.84	0.04	18.76
	98 ≤ C ≤ 100	*Mammuthus primigenius*	8444	0.85	0.06	15.00
	C = 100	*Mammuthus primigenius*	8125	0.86	0.06	14.88

N2	C < 90	*Mammuthus primigenius*	40	0.37	0.13	2.86
	90 ≤ C < 92	*Stenopirates sp.*	12	0.48	0.16	3.00
	92 ≤ C < 94	*Mammuthus primigenius*	23	0.42	0.27	1.53
	94 ≤ C < 96	*Mammuthus primigenius*	35	0.61	0.26	2.33
	96 ≤ C < 98	*Mammuthus primigenius*	57	0.65	0.23	2.85
	98 ≤ C < 100	*Mammuthus primigenius*	179	0.76	0.06	12.79
	98 ≤ C ≤ 100	*Mammuthus primigenius*	4169	0.82	0.05	17.59
	C = 100	*Mammuthus primigenius*	3990	0.82	0.05	17.89
N3	C < 90	*Mammuthus primigenius*	174	0.65	0.07	9.67
	90 ≤ C < 92	*Homo sapiens*	194	0.66	0.27	2.46
	92 ≤ C < 94	*Mammuthus primigenius*	99	0.77	0.08	9.90
	94 ≤ C < 96	*Mammuthus primigenius*	122	0.77	0.08	10.17
	96 ≤ C < 98	*Mammuthus primigenius*	737	0.90	0.04	25.41
	98 ≤ C < 100	*Mammuthus primigenius*	672	0.88	0.05	16.00
	98 ≤ C ≤ 100	*Mammuthus primigenius*	16998	0.83	0.05	17.89
	C = 100	*Mammuthus primigenius*	16326	0.83	0.05	17.98
N4	C < 90	*Theileria cervi*	27	0.28	0.04	6.75
	90 ≤ C < 92	*Mammuthus primigenius*	34	0.67	0.12	5.67
	92 ≤ C < 94	*Mammuthus primigenius*	38	0.95	0.05	19.00
	94 ≤ C < 96	*Reclinomonas americana*	9	0.41	0.18	2.25
	96 ≤ C < 98	*Mammuthus primigenius*	57	0.75	0.08	9.50
	98 ≤ C < 100	*Mammuthus primigenius*	20	0.69	0.10	6.67
	98 ≤ C ≤ 100	*Mammuthus primigenius*	673	0.76	0.09	8.63
	C = 100	*Mammuthus primigenius*	653	0.76	0.09	8.37
N5	C < 90	*Andalucia godoyi*	32	0.24	0.18	1.33
	90 ≤ C < 92	*Reclinomonas americana*	12	0.55	0.14	4.00
	92 ≤ C < 94	*Reclinomonas americana*	6	0.50	0.33	1.50
	94 ≤ C < 96	*Mammuthus primigenius*	21	0.95	0.05	21.00
	96 ≤ C < 98	*Mammuthus primigenius*	76	0.84	0.06	15.20

	98 ≤ C < 100	*Mammuthus primigenius*	141	0.90	0.04	23.50
	98 ≤ C ≤ 100	*Mammuthus primigenius*	2645	0.86	0.05	18.12
	C = 100	*Mammuthus primigenius*	2504	0.85	0.05	17.89
N6	C < 90	*Theileria cervi*	117	0.29	0.22	1.31
	90 ≤ C < 92	*Adoxophyes honmai*	1	0.50	0.50	1.00
	92 ≤ C < 94	*Mammuthus primigenius*	108	0.69	0.06	12.00
	94 ≤ C < 96	*Mammuthus primigenius*	85	0.89	0.03	28.33
	96 ≤ C < 98	*Mammuthus primigenius*	400	0.90	0.05	20.00
	98 ≤ C < 100	*Mammuthus primigenius*	511	0.88	0.05	16.48
	98 ≤ C ≤ 100	*Mammuthus primigenius*	10550	0.82	0.05	16.91
	C = 100	*Mammuthus primigenius*	10039	0.82	0.05	16.93
JK2911	<90	*Homo sapiens*	30452	1.00	0.00	342.16
	≥90-92	*Homo sapiens*	46335	1.00	0.00	46335.00
	≥92-94	*Homo sapiens*	143309	1.00	0.00	1592.32
	≥94-96	*Homo sapiens*	480195	1.00	0.00	2609.76
	≥96-98	*Homo sapiens*	1285504	1.00	0.00	4479.11
	≥98-100	*Homo sapiens*	2129591	1.00	0.00	1964.57
	≥98	*Homo sapiens*	30024129	1.00	0.00	3284.20
	100	*Homo sapiens*	27894633	1.00	0.00	3461.73
Afontova-Gora3	<90	*Homo sapiens*	3685	1.00	0.00	1842.50
	≥90-92	*Homo sapiens*	11268	1.00	0.00	11268.00
	≥92-94	*Homo sapiens*	26312	1.00	0.00	26312.00
	≥94-96	*Homo sapiens*	86303	1.00	0.00	43151.50
	≥96-98	*Homo sapiens*	209270	1.00	0.00	1609.77
	≥98-100	*Homo sapiens*	281458	1.00	0.00	8041.66
	≥98	*Homo sapiens*	332193	1.00	0.00	2460.69
	100	*Homo sapiens*	50735	1.00	0.00	507.35
Villabruna	<90	*Homo sapiens*	1000	0.97	0.02	55.56
	≥90-92	*Homo sapiens*	1256	1.00	0.00	1256.00
	≥92-94	*Homo sapiens*	3002	1.00	0.00	3002.00
	≥94-96	*Homo sapiens*	7263	1.00	0.00	7263.00
	≥96-98	*Homo sapiens*	11837	1.00	0.00	5918.50
	≥98-100	*Homo sapiens*	17228	1.00	0.00	17228.00
	≥98	*Homo sapiens*	193260	1.00	0.00	743.31
	100	*Homo sapiens*	176032	1.00	0.00	677.05

‡ The similarity we used here is 98%.

Table S8. Analysis of One-Way ANOVA with LSD for VMH of different query coverage by use of whole animal mtDNA database based on BLAST search

Query Coverage (C, %)		Mean difference (I-J)	Std. Error	Sig.	95% Confidence Interval Lower Bound	Upper Bound
C < 90	C≥90-92	133.50000	3035.67056	.965	-5984.4920	6251.4920
	C≥92-94	82.87500	2628.96782	.975	-5215.4615	5381.2115
	C≥94-96	98.50000	2628.96782	.970	-5199.8365	5396.8365
	C≥96-98	-18.13889	2579.82391	.994	-5217.4323	5181.1546
	C≥98-100	-57.80556	2579.82391	.982	-5257.0990	5141.4879
	C=100	-9950.58333*	2579.82391	.000	-15149.8768	-4751.2899
C ≥ 90-92	C<90	-133.50000	3035.67056	.965	-6251.4920	5984.4920
	C≥92-94	-50.62500	2628.96782	.985	-5348.9615	5247.7115
	C≥94-96	-35.00000	2628.96782	.989	-5333.3365	5263.3365
	C≥96-98	-151.63889	2579.82391	.953	-5350.9323	5047.6546
	C≥98-100	-191.30556	2579.82391	.941	-5390.5990	5007.9879
	C=100	-10084.08333*	2579.82391	.000	-15283.3768	-4884.7899
C ≥ 92-94	C<90	-82.87500	2628.96782	.975	-5381.2115	5215.4615
	C≥90-92	50.62500	2628.96782	.985	-5247.7115	5348.9615
	C≥94-96	15.62500	2146.54324	.994	-4310.4486	4341.6986
	C≥96-98	-101.01389	2086.06506	.962	-4305.2018	4103.1740
	C≥98-100	-140.68056	2086.06506	.947	-4344.8684	4063.5073
	C=100	-10033.45833*	2086.06506	.000	-14237.6462	-5829.2704
C≥94-96	C<90	-98.50000	2628.96782	.970	-5396.8365	5199.8365
	C≥90-92	35.00000	2628.96782	.989	-5263.3365	5333.3365
	C≥92-94	-15.62500	2146.54324	.994	-4341.6986	4310.4486
	C≥96-98	-116.63889	2086.06506	.956	-4320.8268	4087.5490
	C≥98-100	-156.30556	2086.06506	.941	-4360.4934	4047.8823
	C=100	-10049.08333*	2086.06506	.000	-14253.2712	-5844.8954
C ≥ 96-98	C<90	18.13889	2579.82391	.994	-5181.1546	5217.4323
	C≥90-92	151.63889	2579.82391	.953	-5047.6546	5350.9323
	C≥92-94	101.01389	2086.06506	.962	-4103.1740	4305.2018
	C≥94-96	116.63889	2086.06506	.956	-4087.5490	4320.8268
	C≥98-100	-39.66667	2023.78037	.984	-4118.3280	4038.9947
	C=100	-9932.44444*	2023.78037	.000	-14011.1058	-5853.7831
C ≥ 98-100	C<90	57.80556	2579.82391	.982	-5141.4879	5257.0990
	C≥90-92	191.30556	2579.82391	.941	-5007.9879	5390.5990
	C≥92-94	140.68056	2086.06506	.947	-4063.5073	4344.8684
	C≥94-96	156.30556	2086.06506	.941	-4047.8823	4360.4934
	C≥96-98	39.66667	2023.78037	.984	-4038.9947	4118.3280
	C=100	-9892.77778*	2023.78037	.000	-13971.4391	-5814.1164
	C<90	9950.58333*	2579.82391	.000	4751.2899	15149.8768

C = 100	C≥90-92	10084.08333*	2579.82391	.000	4884.7899	15283.3768
	C≥92-94	10033.45833*	2086.06506	.000	5829.2704	14237.6462
	C≥94-96	10049.08333*	2086.06506	.000	5844.8954	14253.2712
	C≥96-98	9932.44444*	2023.78037	.000	5853.7831	14011.1058
	C≥98-100	9892.77778*	2023.78037	.000	5814.1164	13971.4391

‡ The mean difference is significant at the 0.05 level.

Table S9. Testing of the best method and parameters for ancient mammal species identification using 152 ancient samples

Samples	Similarity Ls (%)	SR	*VMH*	*PoVMH (%)*	*R*	Run
horse1	98 ≤ L< 100	*Equus caballus*	123383	0.917134	38.69019	ERR3223781
		Equus przewalskii	3189	0.023705		
horse2	98 ≤ L< 100	*Equus caballus*	2E+06	0.950806	38.60584	ERR3223830
		Equus przewalskii	49386	0.024629		
horse3	98 ≤ L< 100	*Equus caballus*	16653	0.920818	36.20217	ERR3223780
		Equus przewalskii	460	0.025435		
horse4	98 ≤ L< 100	*Equus caballus*	5E+06	0.956522	37.67438	ERR3223822
		Equus przewalskii	121935	0.025389		
horse5	98 ≤ L< 100	*Equus caballus*	121935	0.939086	32.78285	ERR3223877
		Equus przewalskii	121935	0.028646		
horse6	98 ≤ L< 100	*Equus caballus*	121935	0.939019	55.35135	ERR3225461
		Equus przewalskii	121935	0.016965		
horse7	98 ≤ L< 100	*Equus caballus*	121935	0.947222	39.15539	ERR3225565
		Equus przewalskii	121935	0.024191		
horse8	98 ≤ L< 100	*Equus caballus*	121935	0.955995	40.10522	ERR3225463
		Equus przewalskii	121935	0.023837		
horse9	98 ≤ L< 100	*Equus caballus*	121935	0.955476	41.36269	ERR3225477
		Equus przewalskii	121935	0.0231		
horse10	98 ≤ L< 100	*Equus caballus*	121935	0.953546	39.52482	ERR3225561
		Equus przewalskii	121935	0.024125		
horse11	98 ≤ L< 100	*Equus caballus*	121935	0.958523	42.39734	ERR3225458
		Equus przewalskii	121935	0.022608		
horse12	98 ≤ L< 100	*Equus caballus*	121935	0.9522	38.88561	ERR3225476
		Equus przewalskii	121935	0.024487		
horse13	98 ≤ L< 100	*Equus caballus*	121935	0.948373	39.85849	ERR3225491

		Equus przewalskii	121935	0.023793		
horse14	98 ≤ L< 100	*Equus caballus*	121935	0.956401	33.98592	ERR3225537
		Equus przewalskii	121935	0.028141		
horse15	98 ≤ L< 100	*Equus caballus*	121935	0.948718	40.80882	ERR3225545
		Equus przewalskii	121935	0.023248		
horse16	98 ≤ L< 100	*Equus caballus*	121935	0.941415	33.18182	ERR3225606
		Equus przewalskii	121935	0.028371		
horse17	98 ≤ L< 100	*Equus caballus*	121935	0.9637	41.20896	ERR3225488
		Equus przewalskii	121935	0.023386		
horse18	98 ≤ L< 100	*Equus caballus*	121935	0.932295	36.18605	ERR3225542
		Equus przewalskii	121935	0.025764		
horse19	98 ≤ L< 100	*Equus caballus*	121935	0.943377	46.15	ERR3225599
		Equus przewalskii	121935	0.020442		
horse20	98 ≤ L< 100	*Equus caballus*	121935	0.950538	34.3301	ERR3225462
		Equus przewalskii	121935	0.027688		
horse21	98 ≤ L< 100	*Equus caballus*	121935	0.951361	44.35776	ERR3225492
		Equus przewalskii	121935	0.021447		
horse22	98 ≤ L< 100	*Equus caballus*	121935	0.941972	35.57447	ERR3225585
		Equus przewalskii	121935	0.026479		
horse23	98 ≤ L< 100	*Equus caballus*	121935	0.952488	36.9808	ERR3225596
		Equus przewalskii	121935	0.025756		
horse24	98 ≤ L< 100	*Equus caballus*	121935	0.93993	36.47853	ERR3225475
		Equus przewalskii	121935	0.025767		
horse25	98 ≤ L< 100	*Equus caballus*	121935	0.950752	31.08475	ERR3225577
		Equus przewalskii	121935	0.030586		
horse26	98 ≤ L< 100	*Equus caballus*	121935	0.954054	47.9375	ERR3225592
		Equus przewalskii	121935	0.019902		
horse27	98 ≤ L< 100	*Equus caballus*	121935	0.953944	41.18857	ERR3225605
		Equus przewalskii	121935	0.02316		
horse28	98 ≤ L< 100	*Equus caballus*	121935	0.963466	51.27778	ERR3225551
		Equus przewalskii	121935	0.018789		
horse29	98 ≤ L< 100	*Equus caballus*	121935	0.953433	37.07345	ERR3225555
		Equus przewalskii	121935	0.025717		
horse30	98 ≤ L< 100	*Equus caballus*	121935	0.95687	40.23715	ERR3225560
		Equus przewalskii	121935	0.023781		
horse31	98 ≤ L< 100	*Equus caballus*	121935	0.950773	41.85672	ERR3225478

		Equus przewalskii	121935	0.022715		
horse32	98 ≤ L< 100	*Equus caballus*	121935	0.948468	45.30591	ERR3225536
		Equus przewalskii	121935	0.020935		
horse33	98 ≤ L< 100	*Equus caballus*	121935	0.965977	49.87838	ERR3225538
		Equus przewalskii	121935	0.019367		
horse34	98 ≤ L< 100	*Equus caballus*	121935	0.942813	36.75501	ERR3225589
		Equus przewalskii	121935	0.025651		
horse35	98 ≤ L< 100	*Equus caballus*	121935	0.949457	36.99926	ERR3225493
		Equus przewalskii	121935	0.025662		
horse36	98 ≤ L< 100	*Equus caballus*	121935	0.951002	41.09497	ERR3225604
		Equus przewalskii	121935	0.023142		
horse37	98 ≤ L< 100	*Equus caballus*	121935	0.942701	47.9917	ERR3225608
		Equus przewalskii	121935	0.019643		
horse38	98 ≤ L < 100	*Equus caballus*	121935	0.945722	37.20615	ERR3225479
		Equus przewalskii	121935	0.025418		
horse39	98 ≤ L< 100	*Equus caballus*	121935	0.887955	39.01538	ERR3225612
		Equus przewalskii	121935	0.022759		
horse40	98 ≤ L< 100	*Equus caballus*	121935	0.941393	34.4908	ERR3225662
		Equus przewalskii	121935	0.027294		
horse41	98 ≤ L< 100	*Equus caballus*	121935	0.942593	40.76552	ERR3225464
		Equus przewalskii	121935	0.023122		
horse42	98 ≤ L< 100	*Equus caballus*	121935	0.958932	42.64319	ERR3225573
		Equus przewalskii	121935	0.022487		
horse43	98 ≤ L< 100	*Equus caballus*	121935	0.955928	38.66098	ERR3225613
		Equus przewalskii	121935	0.024726		
horse44	98 ≤ L< 100	*Equus caballus*	121935	0.947002	33.70297	ERR3225518
		Equus przewalskii	121935	0.028098		
horse45	98 ≤ L< 100	*Equus caballus*	121935	0.959322	47.50628	ERR3225591
		Equus przewalskii	121935	0.020194		
horse46	98 ≤ L< 100	*Equus caballus*	121935	0.952703	54.3229	ERR3225597
		Equus przewalskii	121935	0.017538		
horse47	98 ≤ L< 100	*Equus caballus*	121935	0.923412	40.08108	ERR3225519
		Equus przewalskii	121935	0.023039		
horse48	98 ≤ L< 100	*Equus caballus*	121935	0.933681	34.97778	ERR3225480
		Equus przewalskii	121935	0.026694		
horse49	98 ≤ L< 100	*Equus caballus*	121935	0.947834	46.45283	ERR3225587

		Equus przewalskii	121935	0.020404		
horse50	98 ≤ L< 100	*Equus caballus*	121935	0.956725	38.54217	ERR3225557
		Equus przewalskii	121935	0.024823		
horse51	98 ≤ L< 100	*Equus caballus*	121935	0.922841	49.25455	ERR3225556
		Equus przewalskii	121935	0.018736		
horse52	98 ≤ L< 100	*Equus caballus*	121935	0.958762	41.48212	ERR3225455
		Equus przewalskii	121935	0.023113		
horse53	98≤L<100	*Equus caballus*	121935	0.942821	47.0625	ERR3225588
		Equus przewalskii	121935	0.020033		
horse54	98≤L<100	*Equus caballus*	121935	0.885076	38.43103	ERR3225582
		Equus przewalskii	121935	0.02303		
horse55	98≤L<100	*Equus caballus*	121935	0.913616	37.64407	ERR3225670
		Equus przewalskii	121935	0.02427		
horse56	98≤L<100	*Equus caballus*	121935	0.937152	39.26667	ERR3225564
		Equus przewalskii	121935	0.023866		
horse57	98≤L<100	*Equus caballus*	121935	0.921265	36.13924	ERR3225514
		Equus przewalskii	121935	0.025492		
horse58	98≤L<100	*Equus caballus*	121935	0.944616	42.63978	ERR3225611
		Equus przewalskii	121935	0.022153		
horse59	98≤L<100	*Equus caballus*	121935	0.967521	41.0255	ERR3225626
		Equus przewalskii	121935	0.023583		
horse60	98≤L<100	*Equus caballus*	121935	0.971975	43.6	ERR3225506
		Equus przewalskii	121935	0.022293		
horse61	98≤L<100	*Equus caballus*	121935	0.933896	39.26939	ERR3225607
		Equus przewalskii	121935	0.023782		
horse62	98≤L<100	*Equus caballus*	121935	0.969697	35.2	ERR3225505
		Equus przewalskii	121935	0.027548		
horse63	98≤L<100	*Equus caballus*	121935	0.967379	34.4	ERR3225454
		Equus przewalskii	121935	0.028121		
horse64	98≤L<100	*Equus caballus*	121935	0.939493	41.70219	ERR3225457
		Equus przewalskii	121935	0.022529		
horse65	98≤L<100	*Equus caballus*	121935	0.669643	6.617647	ERR3225598
		Equus zebra	121935	0.10119		
horse66	98≤L<100	*Equus caballus*	121935	0.979681	54.06061	ERR3225633
		Equus przewalskii	121935	0.018122		
horse67	98≤L<100	*Equus caballus*	121935	0.973235	45.89634	ERR3225540

		Equus przewalskii	121935	0.021205		
horse68	98≤L<100	*Equus caballus*	121935	0.982582	69.40404	ERR3225548
		Equus przewalskii	121935	0.014157		
horse69	98≤L<100	*Equus caballus*	121935	0.96556	40.25641	ERR3225502
		Equus przewalskii	121935	0.023985		
horse70	98≤L<100	*Equus caballus*	121935	0.964373	30.78431	ERR3225507
		Equus przewalskii	121935	0.031327		
horse71	98≤L<100	*Equus caballus*	121935	0.970729	33.71667	ERR3225513
		Equus przewalskii	121935	0.028791		
horse72	98≤L<100	*Equus caballus*	121935	0.973656	42	ERR3225494
		Equus przewalskii	121935	0.023182		
horse73	98≤L<100	*Equus caballus*	121935	0.970185	36.48485	ERR3225583
		Equus przewalskii	121935	0.026591		
horse74	98≤L<100	*Equus caballus*	121935	0.976619	49.04516	ERR3225486
		Equus przewalskii	121935	0.019913		
horse75	98≤L<100	*Equus caballus*	121935	0.97593	42.88462	ERR3225495
		Equus przewalskii	121935	0.022757		
horse76	98≤L<100	*Equus caballus*	121935	0.949099	26.32353	ERR3225584
		Equus przewalskii	121935	0.036055		
horse77	98≤L<100	*Equus caballus*	121935	0.974083	48.15625	ERR3225610
		Equus przewalskii	121935	0.020228		
horse78	98≤L<100	*Equus caballus*	121935	0.963428	33.30189	ERR3225627
		Equus przewalskii	121935	0.02893		
horse79	98≤L<100	*Equus caballus*	121935	0.980193	59.52293	ERR3225594
		Equus przewalskii	121935	0.016467		
horse80	98≤L<100	*Equus caballus*	121935	0.974407	46.23214	ERR3225509
		Equus przewalskii	121935	0.021076		
horse81	98≤L<100	*Equus caballus*	121935	0.960679	27.5641	ERR3225569
		Equus przewalskii	121935	0.034853		
horse82	98≤L<100	*Equus caballus*	121935	0.971541	42.12766	ERR3225516
		Equus przewalskii	121935	0.023062		
horse83	98≤L<100	*Equus caballus*	121935	0.983387	73.34783	ERR3225510
		Equus przewalskii	121935	0.013407		
horse84	98≤L<100	*Equus caballus*	121935	0.913325	29.72892	ERR3225547
		Equus przewalskii	121935	0.030722		
horse85	98≤L<100	*Equus zebra*	121935	0.404255	1.583333	ERR3225609

		Equus kiang	121935	0.255319		
horse86	98≤L<100	*Equus caballus*	121935	0.96951	39.8481	ERR3225512
		Equus przewalskii	121935	0.02433		
horse87	98≤L<100	*Equus caballus*	121935	0.527027	2.785714	ERR3225664
		Equus grevyi	121935	0.189189		
horse88	98≤L<100	*Equus caballus*	121935	0.974063	51.21212	ERR3225517
		Equus przewalskii	121935	0.01902		
horse89	98≤L<100	*Equus caballus*	121935	0.972337	55.13725	ERR3225496
		Equus przewalskii	121935	0.017635		
horse90	98≤L<100	*Equus caballus*	121935	0.975751	44.64063	ERR3225624
		Equus przewalskii	121935	0.021858		
horse91	98≤L<100	*Equus caballus*	121935	0.974301	41.55769	ERR3225469
		Equus przewalskii	121935	0.023445		
horse92	98≤L<100	*Equus caballus*	121935	0.972828	41.58385	ERR3225566
		Equus przewalskii	121935	0.023394		
horse93	98≤L<100	*Equus caballus*	121935	0.970827	38	ERR3225546
		Equus przewalskii	121935	0.025548		
horse94	98≤L<100	*Equus caballus*	121935	0.974314	43.02466	ERR3225456
		Equus przewalskii	121935	0.022645		
horse95	98≤L<100	*Equus caballus*	121935	0.973563	40.84874	ERR3225525
		Equus przewalskii	121935	0.023833		
horse96	98≤L<100	*Equus caballus*	121935	0.975671	52.24036	ERR3225603
		Equus przewalskii	121935	0.018677		
horse97	98≤L<100	*Equus caballus*	121935	0.973665	51.94937	ERR3225576
		Equus przewalskii	121935	0.018743		
horse98	98≤L<100	*Equus caballus*	121935	0.971831	37.2973	ERR3225641
		Equus przewalskii	121935	0.026056		
horse99	98≤L<100	*Equus caballus*	121935	0.966654	40.58462	ERR3225497
		Equus przewalskii	121935	0.023818		
horse100	98≤L<100	*Equus caballus*	121935	0.977944	53.65	ERR3225524
		Equus przewalskii	121935	0.018228		
horse101	98≤L<100	*Equus caballus*	121935	0.978283	60.53125	ERR3225642
		Equus przewalskii	121935	0.016162		
horse102	98≤L<100	*Equus caballus*	121935	0.963604	35.01613	ERR3225661
		Equus przewalskii	121935	0.027519		
horse103	98≤L<100	*Equus caballus*	121935	0.938462	33.88889	ERR3225472

		Equus przewalskii	121935	0.027692		
horse104	98≤L<100	*Equus caballus*	121935	0.971989	39.65768	ERR3225471
		Equus przewalskii	121935	0.024509		
horse105	98≤L<100	*Equus caballus*	121935	0.975482	45.92967	ERR3225595
		Equus przewalskii	121935	0.021239		
horse106	98≤L<100	*Equus caballus*	121935	0.97333	43.72277	ERR3225659
		Equus przewalskii	121935	0.022261		
horse107	98≤L<100	*Equus caballus*	121935	0.973822	44.89655	ERR3225500
		Equus przewalskii	121935	0.02169		
horse108	98≤L<100	*Equus caballus*	121935	0.955862	49.5	ERR3225550
		Equus przewalskii	121935	0.01931		
horse109	98≤L<100	*Equus caballus*	121935	0.963523	30.34122	ERR3225470
		Equus przewalskii	121935	0.031756		
horse110	98≤L<100	*Equus caballus*	121935	0.971165	42.37097	ERR3225638
		Equus przewalskii	121935	0.022921		
horse111	98≤L<100	*Equus caballus*	121935	0.974638	44.30588	ERR3225663
		Equus przewalskii	121935	0.021998		
horse112	98≤L<100	*Equus caballus*	121935	0.971219	43.22472	ERR3225644
		Equus przewalskii	121935	0.022469		
horse113	98≤L<100	*Equus caballus*	121935	0.970324	38.91	ERR3225616
		Equus przewalskii	121935	0.024938		
horse114	98≤L<100	*Equus caballus*	121935	0.964448	37.36478	ERR3225473
		Equus przewalskii	121935	0.025812		
horse115	98≤L<100	*Equus caballus*	121935	0.950813	25.13	ERR3225498
		Equus przewalskii	121935	0.037836		
horse116	98≤L<100	*Equus caballus*	121935	0.954993	27.83663	ERR3225634
		Equus przewalskii	121935	0.034307		
horse117	98≤L<100	*Equus caballus*	121935	0.95374	22.53488	ERR3225511
		Equus przewalskii	121935	0.042323		
horse118	98≤L<100	*Equus caballus*	121935	0.950094	24.11429	ERR3225653
		Equus przewalskii	121935	0.0394		
horse119	98≤L<100	*Equus caballus*	121935	0.906604	39.22449	ERR3225520
		Equus przewalskii	121935	0.023113		
horse120	98≤L<100	*Equus caballus*	121935	0.925538	35.04639	ERR3225521
		Equus przewalskii	121935	0.026409		
horse121	98≤L<100	*Equus caballus*	121935	0.960063	28.60882	ERR3225529

		Equus przewalskii	121935	0.033558		
horse122	98≤L<100	*Equus caballus*	121935	0.965073	33.81853	ERR3225487
		Equus przewalskii	121935	0.028537		
horse123	98≤L<100	*Equus caballus*	121935	0.958895	30.72131	ERR3225484
		Equus przewalskii	121935	0.031213		
horse124	98≤L<100	*Equus caballus*	121935	0.957306	25.93657	ERR3225485
		Equus przewalskii	121935	0.03691		
horse125	98≤L<100	*Equus caballus*	121935	0.961059	30.75862	ERR3225539
		Equus przewalskii	121935	0.031245		
horse126	98≤L<100	*Equus caballus*	121935	0.965734	34.50467	ERR3225656
		Equus przewalskii	121935	0.027988		
horse127	98≤L<100	*Equus caballus*	121935	0.958629	29.13772	ERR3225645
		Equus przewalskii	121935	0.0329		
horse128	98≤L<100	*Equus caballus*	121935	0.952763	29.09896	ERR3225572
		Equus przewalskii	121935	0.032742		
horse131	98≤L<100	*Equus caballus*	121935	0.968481	36.23708	ERR3225578
		Equus przewalskii	121935	0.026726		
horse140	98≤L<100	*Crocodylus palustris*	121935	1	NA	ERR3225575
horse143	98≤L<100	*Equus caballus*	121935	0.970846	36.22439	ERR3225522
		Equus przewalskii	121935	0.026801		
horse169	98≤L<100	*Equus caballus*	121935	0.921988	43.12987	ERR3225490
		Equus przewalskii	121935	0.021377		
horse171	98≤L<100	*Equus caballus*	121935	0.949495	51.27273	ERR3225617
		Equus zebra	121935	0.018519		
horse174	98≤L<100	*Equus caballus*	121935	0.972859	41.28571	ERR3225614
		Equus przewalskii	121935	0.023564		
horse175	98≤L<100	*Equus caballus*	121935	0.81407	32.4	ERR3225489
		Equus przewalskii	121935	0.025126		
horse181	98≤L<100	*Equus caballus*	121935	0.966389	34.4417	ERR3225523
		Equus przewalskii	121935	0.028059		
horse182	98≤L<100	*Equus caballus*	121935	0.961585	41.78776	ERR3225615
		Equus przewalskii	121935	0.023011		
horse184	98≤L<100	*Equus caballus*	121935	0.966505	29.07663	ERR3225623
		Equus przewalskii	121935	0.03324		
horse198	98≤L<100	*Equus caballus*	121935	0.929595	39.42105	ERR3225640
		Equus przewalskii	121935	0.023581		

horse202	98≤L<100	*Equus caballus*	121935	0.983284	60.51923	ERR3225646
		Equus przewalskii	121935	0.016247		
horse210	98≤L<100	*Equus caballus*	121935	0.940439	36.26415	ERR3225601
		Equus przewalskii	121935	0.025933		
horse213	98≤L<100	*Equus caballus*	121935	0.92644	40.45455	ERR3225602
		Equus przewalskii	121935	0.022901		
horse214	98≤L<100	*Sarcophilus harrisii*	121935	1	NA	ERR3225532
horse216	98≤L<100	*Equus caballus*	121935	0.979137	50.31959	ERR3225531
		Equus przewalskii	121935	0.019458		
horse220	98≤L<100	*Equus caballus*	121935	0.95687	39	ERR3225647
		Equus przewalskii	121935	0.024535		
horse221	98≤L<100	*Equus caballus*	121935	0.954747	37.54228	ERR3225655
		Equus przewalskii	121935	0.025431		
horse222	98≤L<100	*Equus caballus*	121935	0.97517	43.22819	ERR3225639
		Equus przewalskii	121935	0.022559		
horse223	98≤L<100	*Equus caballus*	121935	0.965234	45.82764	ERR3225600
		Equus przewalskii	121935	0.021062		
horse226	98≤L<100	*Equus caballus*	121935	0.949118	43.42945	ERR3225618
		Equus przewalskii	121935	0.021854		
Ancient panda	98≤L<100	*Ailuropoda melanoleuca*	121935	0.34981	1.268966	
		Ursus arctos	121935	0.275665		
Ancient pig	98≤L<100	*Sus scrofa*	121935	0.574132	5.2	
		Osteolaemus tetraspis	121935	0.11041		
Ancient wolf	98≤L<100	*Canis lupus*	121935	0.989246	453.3571	
		Canis latrans	121935	0.002182		

Table S10. Comparisons of species identification under different screening conditions based on deamination induced C-to-T and/or G-to-A changes

Species	The first and last X bases for screening reads with C-to-T and/or G-to-A changes	The top1 species	VMH	PoVMH of top1 species	PoVMH of secondly ranked species	*R*
British aurochs	X=5	*Bos taurus*	662	0.93	0.04	24.52
	X=6	*Bos taurus*	1,667	0.92	0.05	18.32
	X=7	*Bos taurus*	2,127	0.91	0.05	19.34
	X=8	*Bos taurus*	2,583	0.91	0.05	19.87
	X=9	*Bos taurus*	2,591	0.91	0.05	19.63
	X=10	*Bos taurus*	3,020	0.92	0.04	20.54
JK2911	X=5	*Homo sapiens*	462,543	1.00	0.00	5027.63
	X=6	*Homo sapiens*	912,064	1.00	0.00	5011.34
	X=7	*Homo sapiens*	1,303,589	1.00	0.00	3325.48
	X=8	*Homo sapiens*	1,671,700	1.00	0.00	2026.30
	X=9	*Homo sapiens*	2,009,828	1.00	0.00	1792.89
	X=10	*Homo sapiens*	2,353,972	1.00	0.00	2045.15
Direkli5	X=5	*Capra hircus*	290	0.55	0.10	5.27
	X=6	*Capra hircus*	436	0.34	0.33	1.04
	X=7	*Capra hircus*	617	0.37	0.26	1.47
	X=8	*Capra hircus*	786	0.36	0.19	1.87
	X=9	*Capra hircus*	973	0.35	0.18	1.93
	X=10	*Capra hircus*	1,284	0.37	0.17	2.18
Ancient Horse	X=5	*Reclinomonas americana*	3	0.33	0.33	1.00
	X=6	*Andalucia godoyi*	5	0.31	0.19	1.67
	X=7	*Reclinomonas americana*	7	0.23	0.19	1.17
	X=8	*Equus caballus*	78	0.75	0.07	11.14
	X=9	*Equus caballus*	78	0.72	0.08	8.67
	X=10	*Equus caballus*	78	0.70	0.11	6.50
Afontova Gora3	X=5	*Homo sapiens*	33,103	1.00	0.00	848.79
	X=6	*Homo sapiens*	70,337	1.00	0.00	1803.51
	X=7	*Homo sapiens*	101,292	1.00	0.00	2597.23
	X=8	*Homo sapiens*	124,322	1.00	0.00	3108.05
	X=9	*Homo sapiens*	150,521	1.00	0.00	3671.25
	X=10	*Homo sapiens*	173,116	1.00	0.00	4222.35
Villabruna	X=5	*Homo sapiens*	13,816	1.00	0.00	100000.00
	X=6	*Homo sapiens*	26,693	1.00	0.00	635.55

	X=7	*Homo sapiens*	35,187	1.00	0.00	837.79
	X=8	*Homo sapiens*	43,025	1.00	0.00	1024.40
	X=9	*Homo sapiens*	49,501	1.00	0.00	1178.59
	X=10	*Homo sapiens*	57,484	1.00	0.00	1368.67
N1	X=5	*Mammuthus primigenius*	287	0.93	0.04	22.08
	X=6	*Mammuthus primigenius*	625	0.67	0.27	2.50
	X=7	*Mammuthus primigenius*	768	0.70	0.23	3.07
	X=8	*Mammuthus primigenius*	996	0.74	0.19	3.98
	X=9	*Mammuthus primigenius*	1,240	0.76	0.15	4.96
	X=10	*Mammuthus primigenius*	1,389	0.78	0.14	5.56
N2	X=5	*Mammuthus primigenius*	172	0.91	0.04	24.57
	X=6	*Mammuthus primigenius*	335	0.92	0.04	23.93
	X=7	*Mammuthus primigenius*	434	0.90	0.04	25.53
	X=8	*Mammuthus primigenius*	512	0.91	0.03	28.44
	X=9	*Mammuthus primigenius*	551	0.90	0.03	26.24
	X=10	*Mammuthus primigenius*	596	0.90	0.03	27.09
N3	X=5	*Mammuthus primigenius*	467	0.63	0.03	24.58
	X=6	*Mammuthus primigenius*	981	0.76	0.03	23.93
	X=7	*Mammuthus primigenius*	1,474	0.80	0.03	23.40
	X=8	*Mammuthus primigenius*	1,859	0.82	0.04	22.67
	X=9	*Mammuthus primigenius*	2,435	0.84	0.04	23.19
	X=10	*Mammuthus primigenius*	2,838	0.85	0.04	23.45
N4	X=5	*Mammuthus primigenius*	3	0.50	0.33	1.50
	X=6	*Mammuthus primigenius*	23	0.85	0.07	11.50
	X=7	*Mammuthus primigenius*	41	0.87	0.04	20.50
	X=8	*Mammuthus primigenius*	57	0.84	0.06	14.25
	X=9	*Mammuthus primigenius*	57	0.80	0.06	14.25
	X=10	*Mammuthus primigenius*	57	0.79	0.06	14.25
N5	X=5	*Mammuthus primigenius*	70	0.93	0.04	23.33

	X=6	*Mammuthus primigenius*	123	0.95	0.04	24.60
	X=7	*Mammuthus primigenius*	192	0.94	0.03	32.00
	X=8	*Mammuthus primigenius*	283	0.93	0.03	28.30
	X=9	*Mammuthus primigenius*	338	0.92	0.03	28.17
	X=10	*Mammuthus primigenius*	355	0.92	0.03	27.31
N6	X=5	*Mammuthus primigenius*	339	0.95	0.03	28.25
	X=6	*Mammuthus primigenius*	687	0.94	0.04	24.54
	X=7	*Mammuthus primigenius*	892	0.94	0.04	24.11
	X=8	*Mammuthus primigenius*	1,182	0.92	0.04	22.30
	X=9	*Mammuthus primigenius*	1,387	0.93	0.04	23.51
	X=10	*Mammuthus primigenius*	1,759	0.93	0.04	24.10

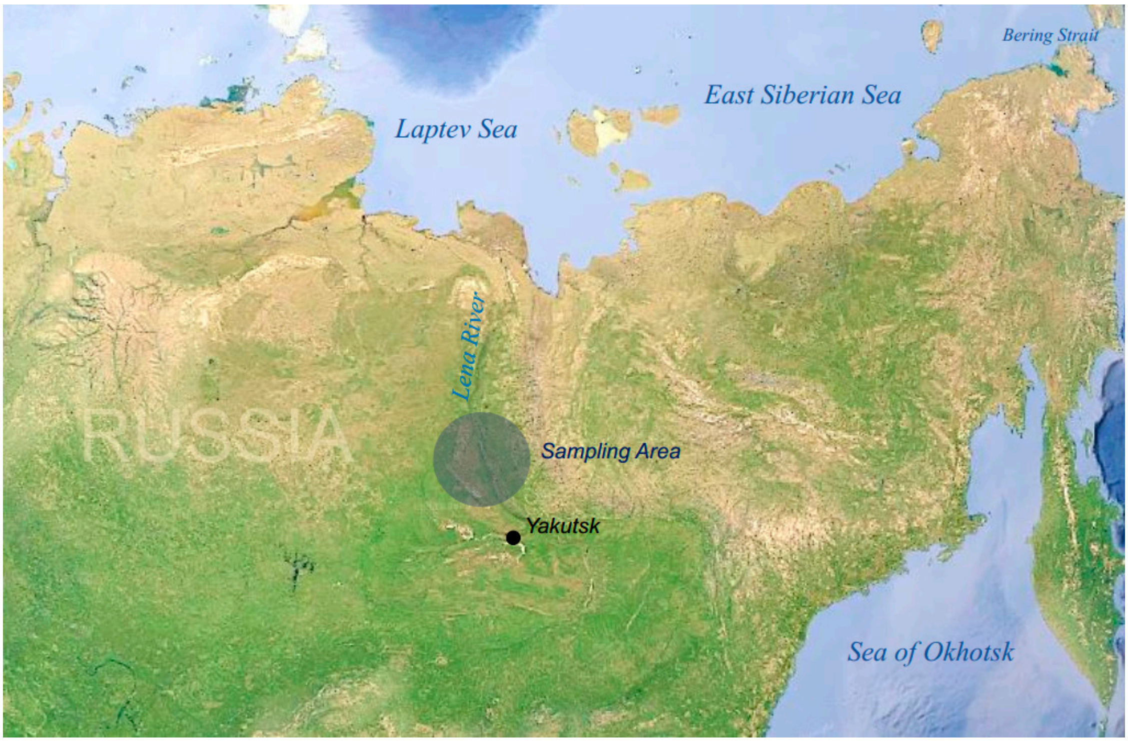

Figure S1. The sampling area of woolly mammoth samples. The dark circle showed the sampling area.

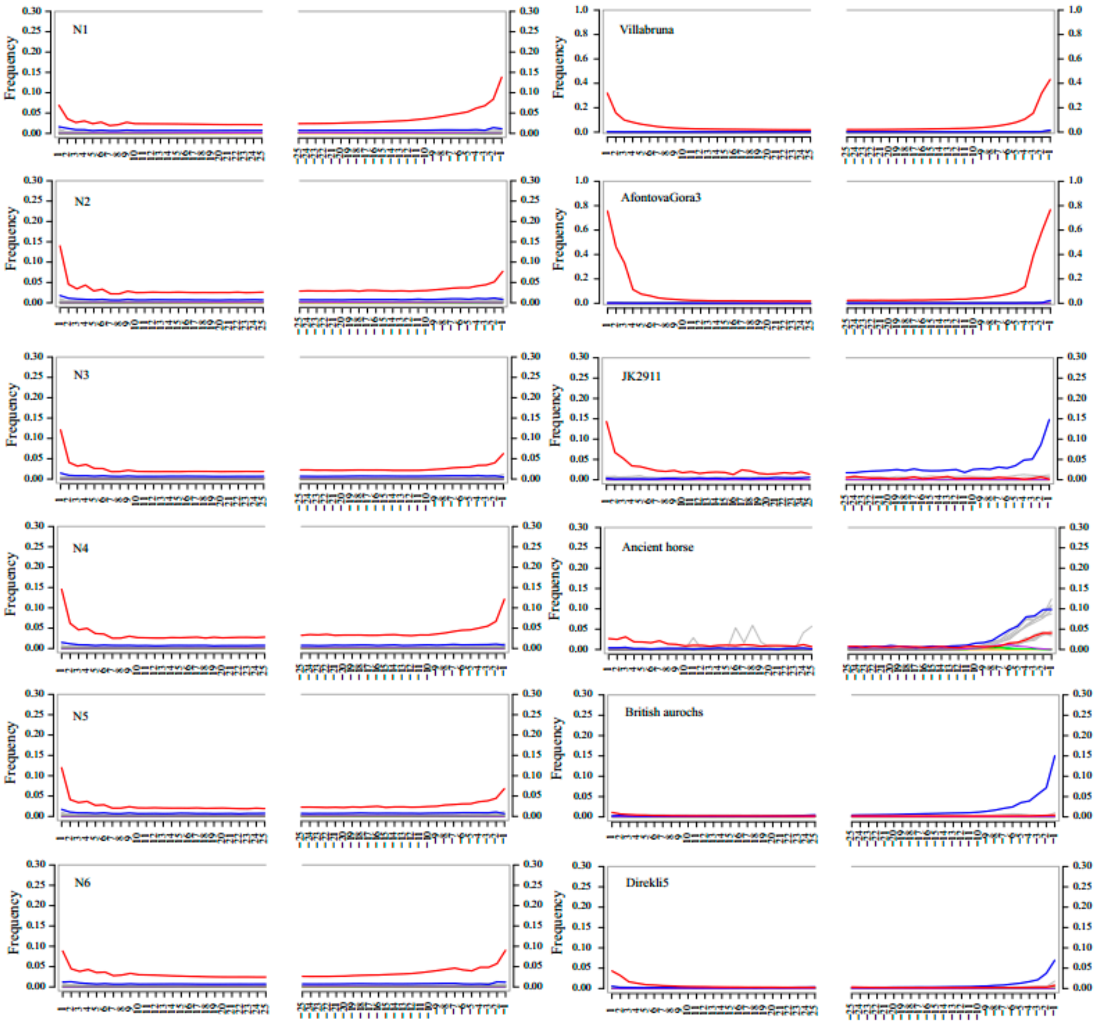

Figure S2. Patterns of cytosine deamination in DNA libraries of 12; samples. The y axis showed the frequencies of nucleotide substitutions, and the x axis showed the distance from 5’ and 3’ ends. Red: C-to-T. Blue: G-to-A.
